# Oligoagars and microbial agents show potential for *Porphyra* disease prevention

**DOI:** 10.1186/s13568-023-01635-7

**Published:** 2023-11-17

**Authors:** Lei Ke, Rui Yang, Qiqin Liu, Yangying Mao, Juanjuan Chen, Qijun Luo, Haimin Chen

**Affiliations:** 1 State Key Laboratory for Managing Biotic and Chemical Threats to the Quality and Safety of Agro-Products, Ningbo University, Ningbo, 315211 Zhejiang China; 2 Collaborative Innovation Center for Zhejiang Marine High-efficiency and Healthy Aquaculture, Ningbo University, Ningbo, 315211 Zhejiang China; 3 School of Marine Science, Ningbo University, No. 169, Qixing South Road, Meishan Bonded Port Area, Ningbo, 315800 Zhejiang China

**Keywords:** *Neoporphyra haitanensis*, Oligoagars, Microbial agents, Microbial community, Disease resistance, Phycosphere

## Abstract

Disease is a major concern in *Porphyra* aquaculture, particularly during the early shell-borne conchocelis (SBC) seedling stage. To explore prevention strategies for *Porphyra* diseases, this study explored the potential of using oligoagars (OA) and microbial agents (MA) to treat SBC of *Neoporphyra haitanensis* in an aquaculture environment. The impact of these treatments on the phycosphere microbial community was analyzed, and the resistance of the treated *Porphyra* conchocelis to the pathogenic bacterium *Vibrio mediterranei* 117-T6 (which causes yellow spot disease) was tested in the lab. Results showed that OA reduced α-diversity while enriching *Rhodobacteriaceae*, and MA increased stability and relative abundance of *Bacteroidetes* (including *Flavobacteria*). Furthermore, compared to the control group, the abundance of pathogenic microorganisms and virulence functional genes decreased while defense-related functional gene abundance increased significantly in the groups treated with OA and MA. Most importantly, the OA and MA treatments improved resistance to Vm117-T6, with survival rates of 70% (OA) and 80% (MA), compared to 15% in the control group. Overall, the findings suggest that OA and MA treatments have great potential for preventing *Porphyra* disease, as they improve phycosphere microorganisms and increase algae resistance to pathogenic bacteria.

**Supplementary Information:**

The online version contains supplementary material available at 10.1186/s13568-023-01635-7.

## Key points

- OA treatment enriched *Rhodobacteraceae* species within phycosphere.
- MA improved microbiota stability, reducing pathogenic microbes and increasing defense genes.
- OA and MA treatments improve the disease status.

**Supplementary Information:**

The online version contains supplementary material available at 10.1186/s13568-023-01635-7.

## Introduction

Algae are a polyphyletic group of mostly photosynthetic organisms with substantial ecological significance and numerous application potentials in the human economy (Karimi et al. 2021). China has a rich history of seaweed cultivation and is currently the world’s largest producer of these macroalgae (Luo et al. 2023). Among the seaweeds cultivated in China, the species *Neoporphyra haitanensis*, a red algae indigenous to the coastal waters of southern China, has been widely farmed, with a production yield of over 70% in the country. In the life cycle of *Pyropia* spp., the conchocelis is a sexual reproduction stage in the life history of *Pyropia* spp. (Chen et al. 2022). It can be divided into two forms: shell-borne conchocelis (SBC) and free-living conchocelis (FLC). Those that grow within shells are referred to as SBC, while the conchocelis that float freely in seawater is called FLC. For *Porphyra* cultivation, SBC sporeling phase is a critical stage that plays a crucial role in ensuring the effective growth and development of the seaweed during subsequent cultivation in the marine environment (Chen et al. 2022).

In recent years, the occurrence of frequent diseases has become a major bottleneck in the development of the *Porphyra* farming industry (Ben 2007; Wang et al. 2020a, b; Ward et al. 2020). While disinfection, extensive water replacement, and shell cleaning can be used to prevent and control diseases during the SBC cultivation stage, chemical use can disrupt the microecological balance of aquaculture systems (Liu et al. 2020). Although a large amount of water change and shell cleaning could alleviate the breeding system’s burden, it cannot effectively improve specific bacterial diseases and may create conditions for the outbreak of pathogenic bacteria. Bacteriophages can be used to increase *Porphyra* survival when infected with pathogenic bacteria (Zhu et al. 2021), but they have a specific inhibitory effect with limited targets. Furthermore, the therapeutic effect may decrease due to the enhanced resistance of pathogenic bacteria to bacteriophages (Hampton et al. 2020). Therefore, it’s necessary to develop more comprehensive and efficient disease prevention and control technologies.

Similar to the rhizosphere of plants, algae cells secrete nutrients outward, creating a specific region around the algae known as the “phycosphere” (Bell and Mitchell 1972; Ramanan et al. 2016). Within this region, microorganisms utilize the extracellular products of algae to grow and supplement their nutrients, while algae also benefit from the microorganisms (Martin et al. 2014). The relationship between phycosphere microbes and cultivated algae is crucial for the growth and health of algae (Wang et al. 2020a, 2022; Yan et al. 2019), understanding the phycosphere microbiome can inform the development of strategies to improve the health and yield of cultivated algae.

In recent years, microbial regulation methods have gained significant attention and application in the aquaculture industry. One such method involves the use of microbial agents (MA) comprising lactic acid bacteria, photosynthetic bacteria, bacillus, yeast, Effective Microorganisms (EM), and biological antimicrobial peptides (Fan et al. 2021). These MA have been widely utilized as feed additives, water quality improvers, and disease prevention and control measures, contributing to the maintenance of ecosystem stability in aquaculture systems. Studies have shown promising results in the application of MA for improving the cultivation of various aquaculture systems, including shrimp larvae and fish (Fan et al. 2021; Hong et al. 2020). Although there are limited reports on the use of MA in seaweed culture, it is feasible to apply this method to regulate microbial populations and control disease occurrence in seaweed cultivation (Li et al. 2022). In fact, some *Porphyra* farmers have already employed MA to manage diseases, indicating the practical implementation of this approach in seaweed cultivation (personal communication with Lei Ke). Therefore, further research is needed to explore the potential of using microbial regulation methods, particularly MA, in seaweed cultivation to enhance production and disease resistance.

Elicitors are a unique category of compounds that have the ability to activate host defense responses (Nurnberger and Kernmerling 2006). One such elicitor class is the oligoagars (OA), which not only enhance resistance to high temperature and decay of *Porphyra* (Chen et al. 2016; Zhu et al. 2012b), but also have the potential to effectively protect it from pathogenic bacteria (Mao et al. 2023). Interestingly, research conducted by Zhu et al. (2012a) reveals that OA also impacts the epiphytic microorganisms residing on *Porphyra*. So, we propose a hypothesis: could the impact of OA stimulation on phycosphere microorganisms be associated with its ability to augment *Porphyra*’s disease resistance?

In the present study, we used OA and MA to treat *Neoporphyra haitanensis* SBC in the breeding environment, using metagenomics to analyzed the changes of phycosphere microorganisms, and infected *Porphyra* conchocelis with the pathogenic bacterium *Vibrio mediterranei* 117-T6 of yellow spot disease (Liu et al. 2019; Yang et al. 2020) in the lab, by combining the survival rate of algae and the changes of microorganisms, we discussed the disease control effects of OA and MA.

## Materials and methods

### Material preparation

#### FLC of *N. haitanensis* ct. ZD-1

The free-living conchocelis (FLC) of *N. haitanensis* ct. ZD-1 was provided by the Key Laboratory of Marine Biotechnology of Zhejiang Province (KLMBZ), Ningbo University, China. FLC with bright red color and healthy condition were selected. The culture medium was NBU-3 (Xu et al. 2020). The culture temperature was 20 °C, pH 8.0 ± 0.2, the salinity of sterile seawater was 25, the photoperiod was 12: 12 h (L:D; light: dark), and the light intensity was 30 μ mol·photons·m^−2^·s^−1^.

#### SBC preparation

The experiment was conducted at Ningbo Yinzhou Jinfeng Aquatic Products Food Co., Ltd (29°32′ N, 121°31′ E). Twelve standardized *Porphyra* sporeling nursery ponds (4 m × 13 m × 0.8 m) were selected randomly. The inoculation time of *Neoporphyra haitanensis* shell-borne conchocelis (SBC) preparation was April 2, 2021. Every 20 shells of *Meretrix meretrix* L. were strung into a string, and the shell strings were hung neatly in the sporeling nursery ponds immersed in seawater. FLC of *N. haitanensis* ct. ZD-1 was broken into about 200-μm fragments by a household blender. About 10–15 g FLC (fresh weight) was sprinkled on the shell strings of about 100 m^2^ area. The ponds were covered with black plastic film for 3 days. The excess items from the shell surface were washed after 10 to 15 days, and then SBC was cultured under natural conditions. After 60 days, the conchocelis was full of shell surface, and the whole shell became dark red, which could be used for the follow-up experiments.

#### OA and MA

The OA was prepared as previously described (Chen et al. 2004). Briefly, 20 g agar powder was added to 1 L pure water and heated until the solution was clarified. Then, the agar solution was degraded with 0.05 mol/L HCl at 90 °C for 2 h. Once the solution was cooled to room temperature, the pH of the solution was adjusted to 6.8 with sodium hydroxide (NaOH) to obtain a 2% OA solution with 2–10 degrees of polymerization. The solution was stored at 4 °C. The MA utilized in this experiment was Effective Microorganisms (EM), which is a novel compound microbial active agent developed by Professor Higa Teruo, a distinguished applied microbiologist from Ryukyu University in Japan (Deng et al. 2020). The EM used in this study was produced by China Shandong Baolai-Leelai Bio-Industrial Group (located in Shandong, China). It appears as a brown translucent liquid with a pH value ranging from 3.5 to 4.5. EM consists of a diverse range of microorganisms, including lactic acid bacteria, butyric acid bacteria, actinobacteria, yeasts, and photosynthetic bacteria. These microbial strains were derived from bacterial clones isolated from various sources such as aquaculture water bodies, sediments, and the surfaces and bodies of aquatic animals. The total viable bacteria count of the EM used in this study was at least 80 × 10^8^ CFU mL^−1^ (CFU: Colony-Forming Units). All of these parameters were provided by China Shandong Baolai-Leelai Bio-Industrial Group.

#### Preparation of Vm 117-T6 (*Vibrio*)

The Vm 117-T6 strain (*Vibrio*) was collected from the China General Microbiological Culture Collection Center under the collection code NO. CGMCC 1.16311. It has been identified as the pathogen responsible for yellow spot disease in *Porphyra* SBC, as reported by Liu et al. (2019) and Yang et al. (2020). The Vm 117-T6 strain, provided by the KLMBZ, was activated and proliferated in tryptic soy broth (TSB) at 28 °C and 110 r/min for 12 h and then centrifuged at 5000×*g* for 10 min at 4 °C. When the bacteria were grown to an OD_600_ of approximately 0.6, the bacterial concentration was adjusted to 10^8^ CFU/mL with boiling-sterilized seawater (filtrated with a 0.22-μm microporous membrane and boiled for 5 min) for subsequent experiments.

#### OA and MA treatment

SBC nursery pond: OA, MA, and MO (MA + OA) treatments were performed on June 12, 2021. Briefly, a 2% OA solution was added according to the water volume of the sporeling nursery pond and evenly sprinkled in the pond to make the final concentration of OA solution 0.1‰. After 1 h, the pond water was replaced. After 48 h, the second OA treatment was carried out with the same steps. MA solution was evenly sprinkled into the nursery ponds at 5 mg/L without any process before sample collection. MO treatment was conducted by adding MA solution into the nursery ponds after OA treatment was completed. Each treatment had three replicates.

FLC was treated with OA, MA, and MO by the same method as SBC nursery ponds for pathogen infection.

### Metagenomics analysis of phycosphere microorganisms

#### Sample collection

After 1 week, the samples of phycosphere microorganisms were collected, including (i) CK (control group without treatment), (ii) OA, (iii) MA, (iv) MO. There were three replicate samples for each group.

The seawater of the nursery pond was maintained under the following conditions: temperature 21–23 °C, pH 8.0–8.3, salinity 20–23, nitrate nitrogen 0.017–0.061 mg/L, and ammonia nitrogen 0.016–0.045 mg/L. Moreover, the conditions did not change with or without the addition of OA, MA, or both (MO).

The sample collection was carried out as previously described (Liu et al. 2020). SBC was collected (about 10 pieces/treatment), the shell surface was washed with sterilized seawater, and 500 mg of conchocelis was scraped from the shell with a sterilized scalpel. The mixture was then filtered through a 0.2-μm polycarbonate membrane (Millipore, Boston, Massachusetts, USA) after being pre-filtered with a 100-μm sterilized nylon mesh to collect phycosphere microorganisms. A total of 12 phycosphere samples were collected and stored at − 80 °C for DNA extraction.

#### DNA extraction, library construction, and metagenomic sequencing

DNA on polycarbonate membrane was extracted using the Power Soil® DNA Kit (MOBIO, Carlsbad, CA, USA). The concentration and purity of extracted DNA were determined with TBS-380 and NanoDrop2000, respectively. DNA quality was checked on 1% agarose gel. DNA samples were stored at − 80 °C. Extracted DNA was fragmented to an average size of about 400 bp using Covaris M220 (Gene Company Limited, Shanghai, China) for paired-end library construction. The paired-end library was constructed using NEXTflex™ Rapid DNA-Seq (Bioo Scientific, Austin, TX, USA). Adapters containing the full complement of sequencing primer hybridization sites were ligated to the blunt end of fragments. Paired-end sequencing was performed on Illumina NovaSeq (Illumina Inc., San Diego, CA, USA) at Majorbio Bio-Pharm Technology Co., Ltd. (Shanghai, China) using NovaSeq Reagent Kits according to the manufacturer’s instructions (www.illumina.com). Sequence data associated with this project have been deposited in the NCBI Short Read Archive database (Accession Number: PRJNA895529).

#### Sequence quality control and genome assembly

The raw reads from metagenome sequencing were used to generate clean reads by removing adaptor-containing sequences and low-quality reads (reads with N bases, a minimum length threshold of 50 bp and a minimum quality threshold of 20) using fastp (Chen et al. 2018) on the free online platform of Majorbio Cloud Platform (cloud.majorbio.com). These high-quality reads were then assembled to contigs using MEGAHIT (Li et al. 2015) (parameters: kmer_min=47, kmer_max=97, step=10). Finally, contigs with a length being or over 300 bp were selected as the final assembling result.

#### Gene prediction, taxonomy, and functional annotation

Open reading frames (ORFs) in contigs were identified using MetaGene (Noguchi et al. 2006). The predicted ORFs with a length being or over 100 bp were retrieved and translated into amino acid sequences using the NCBI translation table.

A non-redundant gene catalog was constructed using CD-HIT (Fu et al. 2012) with 90% sequence identity and 90% coverage. Reads after quality control were mapped to the non-redundant gene catalog with 95% identity using SOAPaligner (Li et al. 2008), and gene abundance in each sample was evaluated.

Representative sequences of non-redundant gene catalog were annotated based on the NCBI NR database using blastp as implemented in DIAMOND v0.9.19 with an e-value cutoff of 1e^−5^ using Diamond (Buchfink et al. 2015) for taxonomic annotations. Cluster of orthologous groups of proteins (COG) annotation for the representative sequences were performed using Diamond (Buchfink et al. 2015) (http://www.diamondsearch.org/index.php, version 0.8.35) against eggNOG database (version 4.5.1) with an e-value cutoff of 1e^−5^. The KEGG annotation was conducted using Diamond (Buchfink et al. 2015) (http://www.diamondsearch.org/index.php, version 0.8.35) against the Kyoto Encyclopedia of Genes and Genomes database (http://www.genome.jp/keeg/, version 94.2) with an e-value cutoff of 1e^−5^. Carbohydrate-active enzymes (CAZy) annotation was conducted using hmmscan (http://hmmer.janelia.org/search/hmmscan) against CAZy database (http://www.cazy.org/) with an e-value cutoff of 1e-5. Virulence factor genes (VFs) annotation was conducted using Diamond (Buchfink et al. 2015) against the VFDB database (http://www.mgc.ac.cn/VFs/) with an e-value cutoff of 1e^−5^. Pathogen annotation for the representative sequences were performed using Diamond (Buchfink et al. 2015) (http://www.diamondsearch.org/index.php, version 0.8.35) against PHI database (http://www.phi-base.org/index.jsp, version 2.2.31) with an e-value cutoff of 1e^−5^.

#### Infection of Vm 117-T6

The infection experiments of FLC were conducted in 250-mL conical flasks containing 100 mL of culture medium and 100 mg of FLC. Each treatment had three replicates. The microscopic characteristics of FLC were observed daily using 0.5% Evan’s blue stain (Yang et al. 2020). The living cells remained red, and the dead cells were dyed blue. The Survival percent represented by the percentage of healthy cells to all cells in more than 20 fields was counted to evaluate the disease resistance efficacy of different treatments.

The infection experiments of SBC were carried out in cuboid plastic boxes (0.3 m × 0.05 m × 0.05 m). The SBC and seawater retrieved from the above treatment ponds were added to each cuboid container. Vm 117-T6 medium solution and seawater were added to the cuboid box at a ratio of 1:1. The shell color and characteristics were photographed. Each treatment had three replicates.

#### Data analysis and statistics

Alpha-diversity indices were computed using Phyloseq (Mcmurdie et al. 2013). One-way analysis of variance (ANOVA) was applied to test the differences in microbial community α-diversity of phycosphere under different treatment conditions using SPSS 22. Community distance matrices based on the Bray–Curtis dissimilarity index were estimated using vegan (Dixon 2003) and visualized by non-metric multidimensional scaling (NMDS). Permutational analysis of variance (PERMANOVA) and analysis of similarity (ANOSIM) tests were performed in vegan (Dixon 2003) to evaluate changes in microbiome structure and composition across treatments. Stability of microbial communities was evaluated by calculating the average variation degree (AVD) index (Xun et al. 2021). Differential abundance analysis of taxonomic groups (log fold change ≥ |2|, Benjamini–Hochberg (BH)-adjusted *P*-value ≤ 0.05) was performed using Phyloseq-DESeq (Mcmurdie et al. 2014) through pairwise comparisons between control and treated samples.

Statistical significance of the different treatment groups was tested using one-way ANOVA with a significance level of *P* < 0.05, utilizing SPSS 22 and Prism 9 software.

## Results

### OA lowered microbial diversity, while MA improved microbial stability

**Fig. 1:**
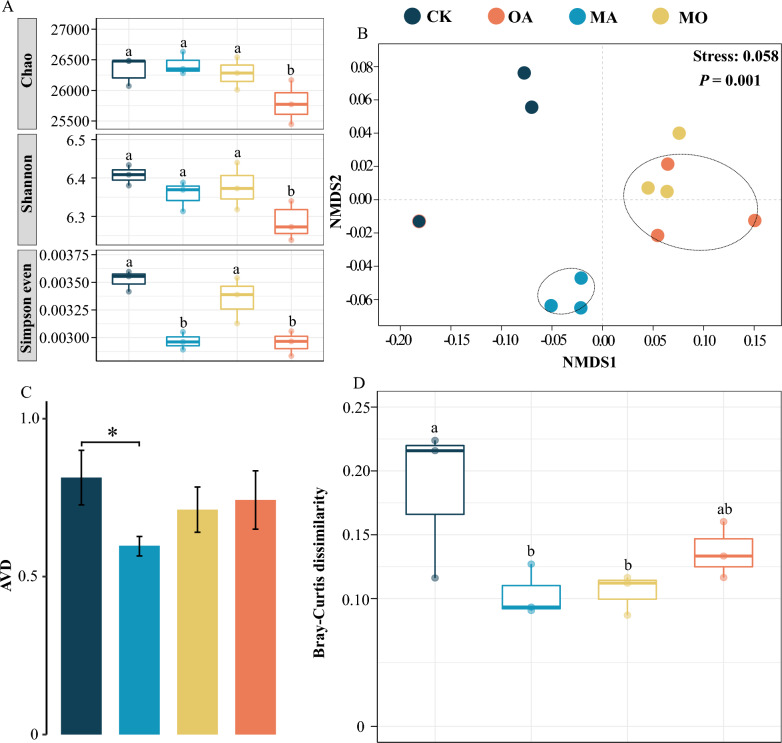
Shows the comparison results of microbial community diversity analysis in different groups. **A** Changes in α-diversity indices. **B** NMDS analysis based on Bray–Curtis distance. **C** Changes in AVD indices. **D** Beta diversity distance analysis based on Bray–Curtis distance

As shown in Fig. 1A, the α-diversity index of the phycosphere microbial community was significantly decreased by OA treatment, while MA treatment had no significant effect. However, the NMDS plot manifested significant changes in the phycosphere microbiota during the treatment process (*P* = 0.001), despite the lack of statistically significant differences in microbial communities between the CK and different treatment groups.

Remarkably, the samples from the MA group clustered together, forming a smaller group, as shown in Fig. 1B, suggesting a more stable phycosphere microbial community in the MA treatment group. This observation is supported by the lower AVD values found in the MA group, as higher stability of microbial communities is indicated by lower AVD values (Xun et al. 2021), as displayed in Fig. 1C.

It is worth noting that samples in the OA group and MO group were close proximityto each other in the NMDS plot (Fig. 1B). Further analysis indicated that the R_OA VS. MO_ < R_MA VS. MO_, meaning that microbial communities differed greatly between the MO and MA treatment groups, while the difference was smaller compared to the OA treatment group (Table 1).

**Table 1:** Analysis of similarity of microbial community structure based on Bray–Curtis distance

	PERMANOVA		ANOSIM	
	R^2^	*P*	R	*P*
ALL	0.572	**0.001**	0.676	**0.001**
CK VS. OA	0.507	0.1	0.703	0.098
CK VS. MA	0.404	0.1	0.556	0.098
CK VS. MO	0.511	0.1	0.667	0.098
MO VS. OA	0.214	0.1	0.370	0.201
MO VS. MA	0.558	0.1	1	0.098

*P* < 0.05 means a significant difference, *P* < 0.01 means a very significant difference

### OA treatment enriched *Rhodobacteraceae* species within phycosphere

**Fig. 2:**
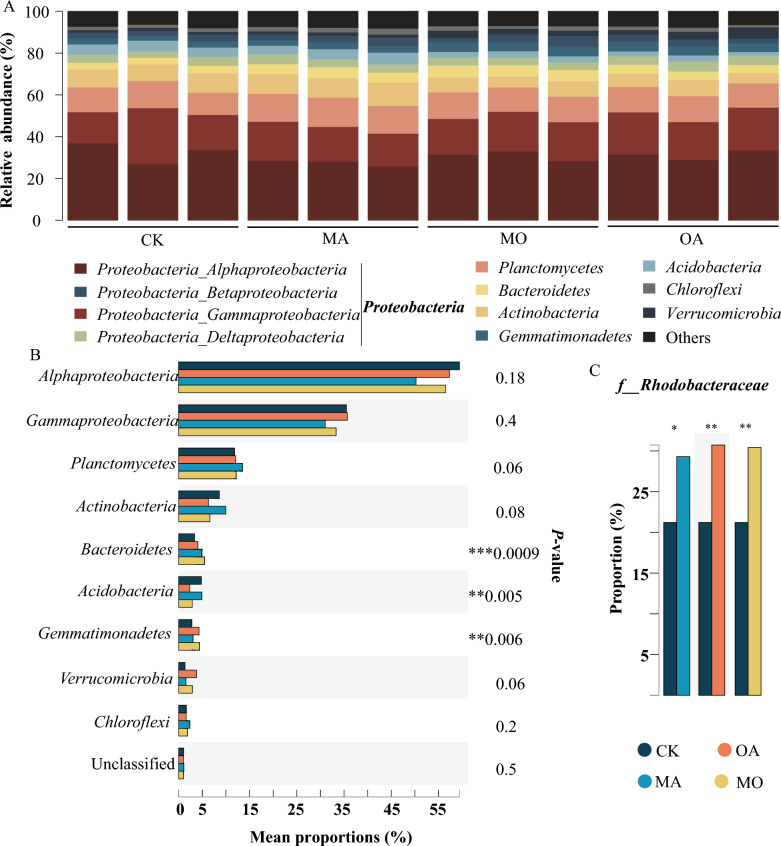
Microbial community composition analysis. **A** Dominant phyla/*proteobacteria* classes with a relative abundance > 1% in each group. **B** STAMP analysis of significant differences in the top 10 phyla/*proteobacteria* classes between different groups. **C** Significant differential families with the highest abundance within the phylum *Proteobacteria* (*P < 0.05, **P < 0.01, ***P < 0.001)

As shown in Fig. 2A, *Proteobacteria* (including *Alpha-*, *Beta-*, *Gamma-*, and *Delta-Proteobacteria*), *Plactomyctes*, and *Bacteroidetes* are dominant phyla in the phycosphere, further analysis showed significant changes in *Bacteroidetes* under different treatment conditions (Fig. 2B), and the most abundant family in *Proteobacteria*, *Rhodobacteraceae*, also showed significant changes (Fig. 2C).

**Fig. 3:**
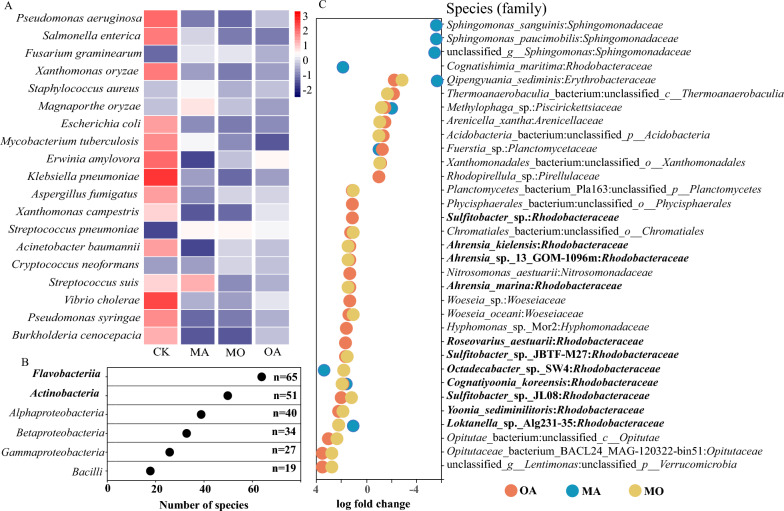
Analysis of differentially abundant species. **A** Heatmap of relative abundance of Pathogen in the PHI database, where red indicates an increase in relative abundance and blue indicates a decrease in relative abundance. **B** Number of differentially abundant species (classified at the class level) produced by MA treatment compared to the control group. **C** Differences in species produced by OA, MA, and MO treatments compared to CK in phycosphere microbiota (abundance top 100) (log fold change ≥ |2|, BH adjusted *P* ≤ 0.05)

Through Phyloseq-DESeq analysis, it was discovered that in the abundance top 100 of the phycosphere microbiota, OA and MO treatments significantly upregulated numerous species belonging to the *Rhodobacteraceae*, including *Ahrensia* species (eg. *Ahrensia_kielensis*, *Ahrensia_*sp._13_GOM-1096m, *Ahrensia_marina*); *Roseovarius* species (e.g., *Roseovarius_aestuarii*), *Octadecabacter* species (e.g., *Octadecabacter_*sp._SW4), and *Sulfitobacter* species (e.g., *Sulfitobacter_*sp._JL08) (log fold change ≥ |2|, BH-adjusted *P* ≤ 0.05; Fig. 3C) compared to the CK group. Further analysis by Phyloseq-DESeq revealed that *Flavobacteria* and *Actinobacteria* were the most upregulated taxa in response to MA treatment (Fig. 3B).

In PHI-base, a total of 207 pathogens have been annotated (Additional file 1: Table S1). The heatmap of the top 20 abundant pathogens relative abundance showed that, compared to the CK group, the relative abundance of MA, MO, and OA treatment groups was lower (Fig. 3A).

### Impact of treatments on phycosphere microbial community functional genes

**Fig. 4:**
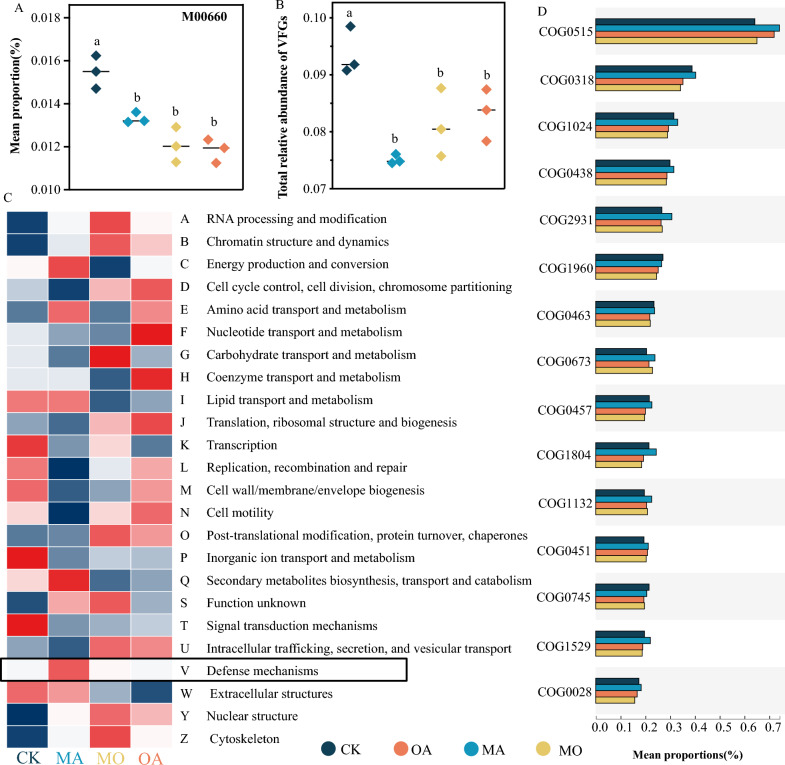
Functional analysis. **A** Average relative abundance of the M00600 gene, which is a KEGG Module associated with disease, in each group. **B** Total relative abundance of VFs in each group. **C** Heatmap showing relative abundance changes in COG function across each group. **D** Significantly different COGs (top 15 by relative abundance) under different treatment conditions

Our metagenomic analysis results showed significant differences in the functional composition of phycosphere microorganisms, including KO (KEGG Orthology), COG, CAZy, and VFs, among different treatment groups (*P* < 0.05, see Additional file 1: Fig. S1). Among them, the KEGG module M00660 (KEGG Pathway) is related to diseases, and we found that its abundance was highest in the CK group, while significantly lower in the OA, MA, and MO groups (*P* < 0.05, Fig. 4A). We annotated a total of 1179 VFs in the VFDB database (see Additional file 1: Table S2), among which the total relative abundance of VFs was highest in the CK group, while significantly lower in the OA, MA, and MO groups (P < 0.05, Fig. 4B). The relative abundance heat map of COG functions is shown in Fig. 4C, and we found that the relative abundance of defense mechanisms was highest in the MA treatment group.

We also listed the COG entries with significant differences (top 15 abundance) in each treatment group (Fig. 4D) and found that most of them were higher in the MA group than in the CK group. These COGs may play important roles in the growth, reproduction, and defense of algae-associated microorganisms. For example, COG0318 plays an important role in DNA damage repair, cell division, and homologous chromosome recombination (Poidevin et al. 2012), COG0463 affects maintaining growth stability (Andersen et al. 2018), COG0515 may function as a signaling molecule or be associated with cell growth and differentiation, stress response, DNA protection, etc. (Kumar et al. 2018), and COG0745 may play an important role in maintaining growth and metabolism (Ma et al. 2017).

### OA and MA treatments improve the disease status

**Fig. 5:**
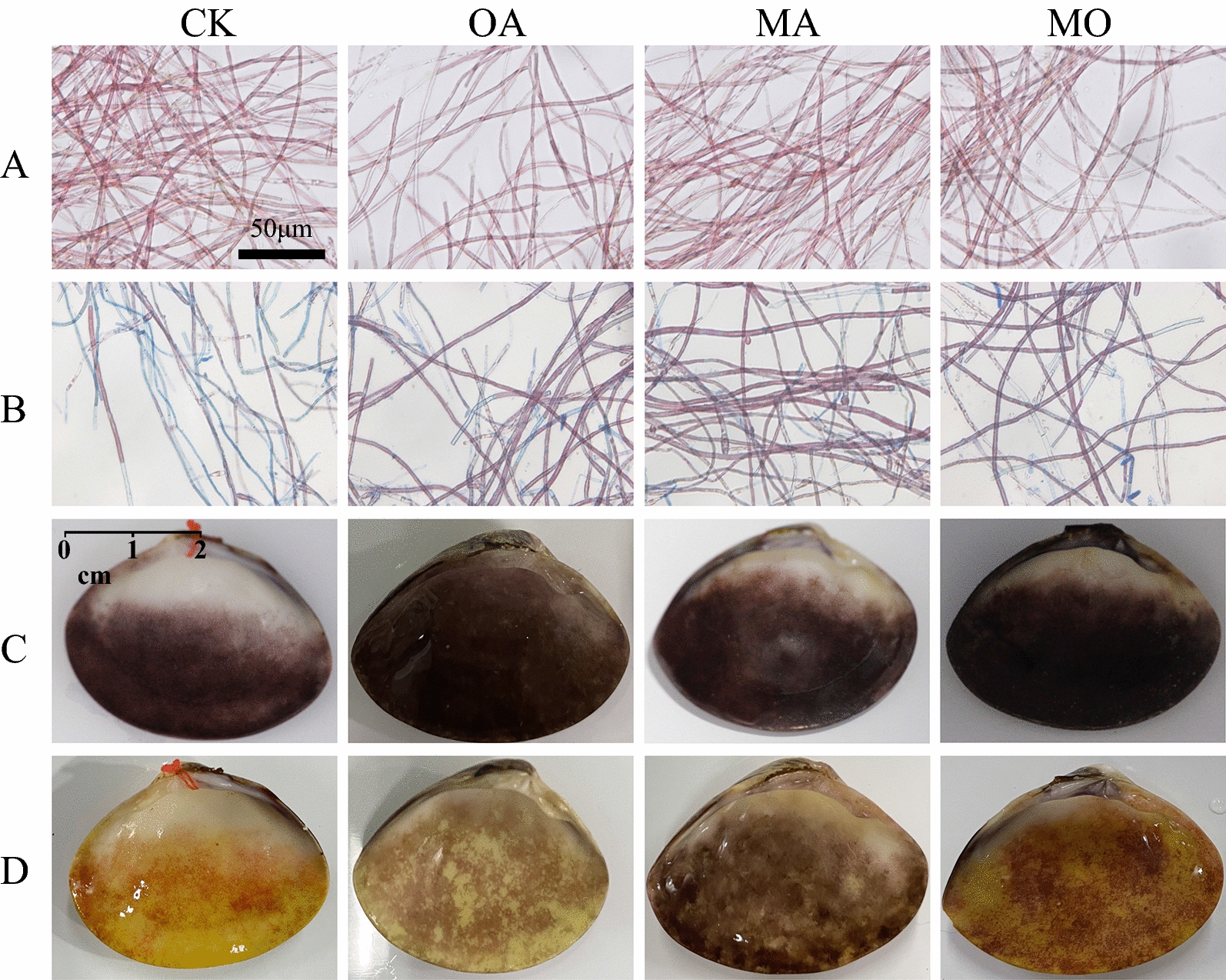
Observation of Vm 117-T6 infected FLC (24 h) and SBC (20 days). *CK* control group, *OA* OA-treated conchocelis, *MA* MA-treated conchocelis, *MO* MO-treated conchocelis; **A** FLC without Vm 117-T6; **B** FLC + Vm 117-T6; **C** SBC without Vm 117-T6; **D** SBC + Vm 117-T6. **A**, **B** bar = 50 μm; **C**, **D** bar = 1 cm

**Fig. 6:**
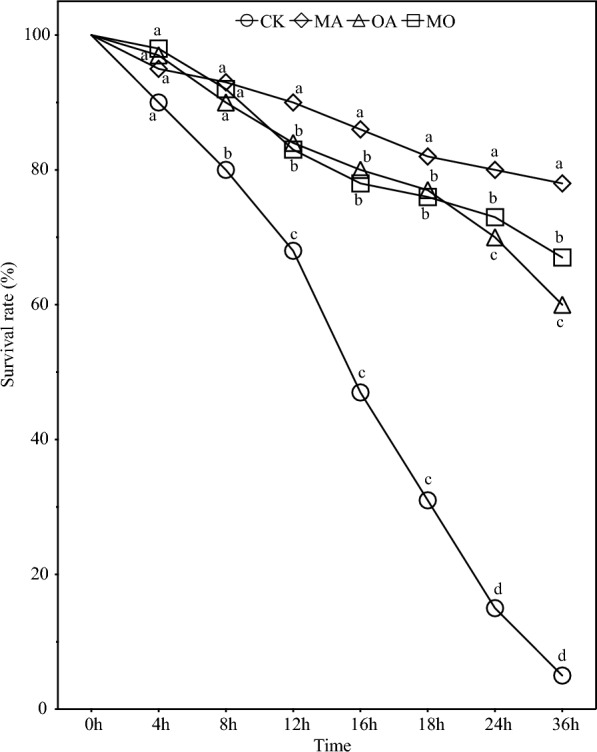
Survival rate of FLC infected by Vm 117-T6 in the CK, MA, OA, and MO groups. The different letters indicate significant difference between treatments (*P* < 0.05)

Overall, OA and MA treatments showed significant improvements in the disease status of *N. haitanensis* conchocelis infected by Vm 117-T6 (Figs. 5 and 6). After being infected by Vm 117-T6 for 24 h, the survival rate of the FLC treated with MA was the highest at 80%, followed by OA and MO treatments at 70%. In contrast, the control group only had a survival rate of 15%, which was significantly lower than the treatment groups (*P* < 0.05).

Similarly, the condition of SBC infected by Vm 117-T6 also showed the same trend. After being infected by Vm 117-T6 for 20 days, the area of yellow spots in SBC treated with MA was the smallest, followed by OA and MO treatment groups. In contrast, the area of yellow spots in SBC of the control group was the largest, almost completely bleached.

## Discussion

### OA enriched probiotics related to algae in the phycosphere

According to reports, OA induces defense reactions in algae, producing a variety of defense related compounds, including reactive oxygen (H_2_O_2_), NO, phenols, and various volatile components, many of which have bactericidal effects (Pohnert 2004), it could be inferred from this that OA indirectly impacts phycosphere microorganisms through algal activities (Mao et al. 2023). The present study revealed that the application of OA significantly reduced the α-diversity index of the phycosphere microbial community, which is in line with earlier findings by Zhu et al. (2012a). Notably, we also observed that the microbial community β-diversity was higher in the OA treatment group (i.e. the community structure was dispersed among samples), which may be attributed to the re-enrichment and colonization of phycosphere microorganisms by algae after OA stimulation.

In algae research, *Rhodobacteraceae* has been widely studied as a probiotic microbiota that resistant algal pathogens (Geng and Belas 2010; Luo and Moran Mary 2014). For example, Dogs et al. (2017) found that *Sulfitobacter* and *Octadecabacter* related to *Fucus spiralis* can protect algae from harmful bacteria and other microorganisms. In this study, OA treatment effectively enriched the phycosphere with *Rhodobacteriaceae* taxonomics such as *Ahrensia*, *Roseovarius*, *Octadecabacter*, and *Sulfitoactor*, and most of them were related to *Roseobacter* (Wu et al. 2014), which is known for its positive role in the defense process of algae (Alavi et al. 2001).

Consequently, we believe that in addition to stimulating the immune defense response of algae (Chen et al. 2016; Du et al. 2018; Mao et al. 2023; Zhu et al. 2012a, b), OA can also enrich related probiotics in the phycsophere. These probiotics inhibited the infection of Vm117-T6 and enhanced the defense ability of the phycsophere microorganisms, thereby maintaining a high survival rate of *N. haitanensis* conchocelis.

### MA increased phycosphere microbial community stability and defense capabilities

The MA utilized in our study is EM bacteria, which is known for its high survival and reproductive rate, allowing it to quickly and stably occupy ecological niches and become a dominant microflora (Khouadja et al. 2017). *Actinobacteria*, as one of the major components of EM bacteria, was found to be significantly enriched in the phycosphere of the MA treatment group. This suggests that upon MA application into the seaweed cultivation system, advantageous microbial groups, such as *Actinobacteria*, may rapidly colonize the phycosphere and form a dominant bacterial community, this will creates an environment unfavorable to the growth of pathogenic microorganisms. Moreover, EM bacteria will also secretes special substances that inhibit the growth of harmful strains (Deng et al. 2020), as supported by our results, which showed lower pathogen abundance in the phycosphere of the MA-treated group compared to the control group. But overall, our results indicate that MA treatment did not have a significant impact on the diversity of phycosphere microorganisms, this result can be attributed to the fact that under normal circulations, macroalgae can attract microbial members, which is beneficial to the algae to colonize and reproduce on its surface (Egan et al. 2013; Liu et al. 2020). This conclusion is consistent with the neutral attributes of MA, such as being green and pollution-free.

Xun et al. (2021) used the AVD index to evaluate the stability of microbial communities. and the results showed that MA treatment led to lower AVD values for phycosphere microbiota. Generally, lower AVD values indicate higher community stability, suggesting that MA treatment increased stability in the phycosphere microbiota community. Furthermore, the taxonomic group with the largest number of significantly up-regulated species was *Flavobacteriia*, which are mostly beneficial microorganisms in marine aquaculture systems and play a vital role in maintaining colony balance (Keller-Costa et al. 2021; Wang et al. 2015).

Combined with the fact that MA treated *N. haitanensis* conchocelis had a higher survival rate under Vm117-T6 infection, it can be concluded that MA treatment made the phycsophere microbiota more stable, while the enriched microbial community in the phycsophere community played a positive defensive role with the microbial community in MA, resulting in higher survival rates of algae.

### OA had a greater effect on phycosphere microbes when combined with MA

Although both MA and OA improved the composition of microorganisms in the phycsophere, MO treatment induced changes similar to those of OA, suggesting that OA played a more crucial role than MA in modulating the phycosphere microorganisms. This may be due to the ability of OA as an elicitor to induce the production of various defense related compounds in algae (Pohnert 2004), which have a broad-spectrum inhibitory effect on various microorganisms in the environment, whether beneficial or harmful. As a result, the inability of the microbial community in MA to colonize in the algae, reducing the protective efficiency of MA and limiting its role in phycosphere modulation.

Previous studies have indicated that OA treatment has the most significant impact on the algal environment within 1–3 h (Du et al. 2018; Zhu et al. 2012a). However, in this experiment, MA was added immediately after the OA treatment was completed. Therefore, the combined processing effect of OA and MA was not reflected. To overcome this, in future *Porphyra* cultivation, we recommend that MA treatment should be carried out 3 h after the completion of OA treatment, which could improve the combined effect of OA and MA treatment on phycosphere modulation.

### Microbial intervention may prevent and control algal disease

The interaction between susceptible hosts, toxic pathogens, and the environment can trigger disease development, and the disease can be prevented if any of the “disease triangle” factors are eliminated (Scholthof 2007). For example, reducing the susceptibility of the host or altering the environmental adaptability of the pathogen can decrease disease incidence (Agrios 2005). Human intervention has been integrated into the disease triangle system to form a disease prevention and control tetrahedron (Andrade-Piedra et al. 2008). People can achieve the goal of disease prevention and treatment by regulating the environment, strengthening the host, inhibiting pathogens, and other ways to achieve the health of the artificial cultivation system. In this study, Both OA and MA can improve microecology of *N. haitanensis* SBC aquaculture system, and both treatments significantly approved the resistance of *N. haitanensis* to Vm 117-T6. These findings pave the way for applying OA and MA to control the diseases of *N. haitanensis* through micro-regulation, providing a valuable reference for the improvement of seaweed culture in the future. Furthermore, in terms of economic effect, the cost of the OA protection method used in this study was only 1.5–3 CNY/100 m^2^ each time, while it was 10–15 CNY/100 m^2^ each time for the MA protection method. Overall, the use of OA and MA could have significant implications for the sustainable and profitable cultivation of *N. haitanensis* and other commercially important algae species.

### Supplementary Information

**Additional file 1: Figure S1.** Analysis of functional structure changes in phycosphere microbial communities. **Table S1.** Pathogen abundance table annotated in the PHI database. **Table S2.** Abundance of VFGs annotated in the VFDB database.

## Data Availability

Sequence data associated with this project have been deposited in the NCBI Short Read Archive database (Accession Number: PRJNA895529).
