# Supplementary material for: Oligoagars and microbial agents show potential for Porphyra disease prevention
Source: AMB Express. 2023 Nov 17;13:128. doi: 10.1186/s13568-023-01635-7 (PMC10656394; doi:10.1186/s13568-023-01635-7)
Supplement: Supplementary file 1 — Additional file 1: Figure S1. Analysis of functional structure changes in phycosphere microbial communities. Table S1. Pathogen abundance table annotated in the PHI database. Table S2. Abundance of VFGs annotated in the VFDB database. [file 13568_2023_1635_MOESM1_ESM.pdf]

Additional file 1

Journal name: AMB Express

Manuscript Title: Oligoagars and Microbial Agents Show Potential for *Porphyra* Disease Prevention

Lei Ke[1,3], Rui Yang[1, 2, 3]*, Qiqin Liu[1, 2, 3], Yangying Mao[1,3], Juanjuan Chen[1, 2, 3], Qijun Luo[2, 3], Haimin Chen[1, 2, 3]

1. State Key Laboratory for Managing Biotic and Chemical Threats to the Quality and Safety of Agro-products, Ningbo University, Ningbo, 315211, Zhejiang, China

2. Key Laboratory of Marine Biotechnology of Zhejiang Province, Ningbo University, Ningbo, 315211, Zhejiang, China

3. School of Marine Science, Ningbo University, No. 169, Qixing South Road, Meishan Bonded Port Area, Ningbo, 315800, Zhejiang, China

*Correspondence: No. 169, Qixing South Road, Meishan Bonded Port Area, Ningbo University, School of Marine Science, Ningbo, Zhejiang, 315800, China

Corresponding author: R. Yang

E-mail: yangrui@nbu.edu.cn

Tel.: 86-13738432

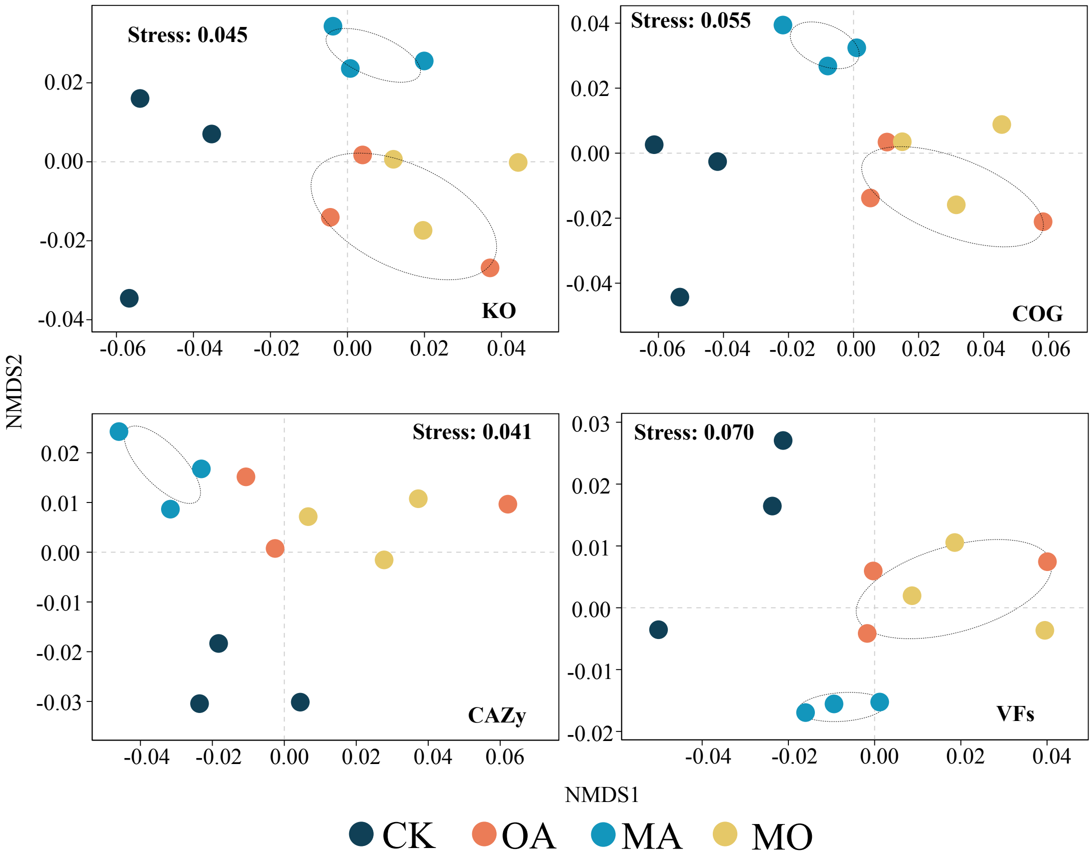

Figure S1 Analysis of functional structure changes in phycosphere microbial communities.

Table S1 Pathogen abundance table annotated in the PHI database

**Pathogen**	CK1	CK2	CK3	MA1	MA2	MA3	MO1	MO2	MO3	OA1	OA2	OA3
Acinetobacter baumannii	136614	118888	132992	105244	106654	115902	111406	153240	128572	100912	128778	123124
Acinetobacter nosocomialis	3138	3800	3424	2218	2342	2736	2850	3932	3138	2482	3018	3188
Actinobacillus pleuropneumoniae	12300	11060	12132	12430	12468	13616	11528	13864	13152	9604	13790	12068
Aeromonas hydrophila	1746	1764	1774	1666	1438	1948	1508	1686	1650	1172	1608	1418
Aeromonas salmonicida	570	510	684	554	616	718	588	922	884	472	648	720
Agrobacterium tumefaciens	2278	1906	2320	1748	1796	1944	1768	2416	1842	1560	1852	2038
Agrobacterium vitis	20200	17288	21622	18826	18942	21906	18946	22380	21144	16528	21798	20294
Alternaria alternata	13688	8210	11884	9112	9066	10270	9116	11158	9936	7640	9814	9206
Alternaria brassicicola	1548	1210	1316	1288	1358	1558	1112	1534	1298	1294	1652	1526
Alternaria longipes	1546	1380	1624	1362	1674	1756	1748	2138	2086	1538	1852	1674
Alternaria solani	92	84	158	68	62	126	74	96	100	50	116	98
Arthrobotrys oligospora	1736	1612	1786	1842	1830	1976	1644	1752	1552	1114	1718	1342
Aspergillus flavus	2858	1868	3562	2152	1954	2366	2208	3404	2524	2096	2430	2698
Aspergillus fumigatus	154660	120500	148606	119230	122356	132732	132890	167564	143214	111330	142996	145612

Aspergillus nidulans	402	300	382	466	438	636	400	398	454	406	542	444
Bacillus anthracis	924	762	980	880	892	966	1182	1180	976	792	1394	1860
Bacillus cereus	34372	29158	34624	28850	27990	30264	28068	37674	32032	24604	31318	30824
Beauveria bassiana	31902	27404	30920	27414	27964	29946	30216	37372	31738	24348	31614	32638
Bipolaris maydis	10864	8824	10532	9462	9216	10382	7848	10096	9144	7304	9620	8638
Bipolaris oryzae	22	48	32	70	54	34	30	44	28	18	64	64
Bipolaris sorokiniana	638	670	612	546	474	660	534	602	638	572	624	516
Bipolaris zeicola	5976	4488	5254	4492	4550	5304	4492	5610	4838	3740	4556	4702
Blumeria graminis	214	194	210	256	424	222	1132	1038	634	456	688	1322
Bordetella bronchiseptica	15400	13750	17242	15022	15300	16510	14434	18092	17590	12988	16754	15306
Bordetella pertussis	5230	4894	6284	3940	4452	4824	4338	5604	5076	3714	4792	4158
Borreliella burgdorferi	60594	52646	64836	58214	58968	65214	58382	71104	66494	50664	67256	62324
Botrytis cinerea	14982	12706	15460	12224	13490	13628	14712	18260	14932	11154	14924	14230
Brucella abortus	71358	59872	71616	61428	61208	66992	60146	81464	70634	54296	68240	67114
Brucella melitensis	22452	17250	22112	15742	17288	18014	16542	22880	18786	16110	18534	18742
Brucella suis	470	386	466	522	614	532	510	944	442	546	612	552
Burkholderia cenocepacia	109652	95636	108256	85462	86004	96932	87132	121962	105540	83226	102894	99528

Burkholderia contaminans	3540	2244	3826	2296	2456	2618	2292	3136	2398	2162	2094	1864
Burkholderia glumae	48974	45020	49190	42572	43408	47130	43650	56346	50664	39630	51782	50516
Burkholderia pseudomallei	43504	36864	44140	37986	38856	43416	34482	47882	42550	31710	43264	39570
Burkholderia thailandensis	1372	1086	1336	1250	1446	1620	1464	1724	1682	1300	1542	1802
Bursaphelenchus xylophilus	3390	2932	3058	2582	2582	2522	3094	3842	2876	2498	3224	3190
Campylobacter jejuni	31240	27712	30352	23056	22792	25856	23814	32828	27788	22740	27638	26606
Candida albicans	56066	53004	57074	52534	57428	60248	59230	69770	61660	44552	60014	61074
Candida dubliniensis	0	0	10	2	16	6	0	2	24	38	0	28
Candida glabrata	4582	4702	5054	3770	4236	4148	5196	5890	4798	3426	4072	4996
Candida parapsilosis	482	928	506	564	816	654	990	1224	984	550	1076	1326
Candida tropicalis	318	200	220	266	430	320	364	412	368	238	314	488
Cercospora kikuchii	258	196	448	256	306	388	246	230	302	246	308	188
Cercospora nicotianae	866	670	720	476	666	914	620	840	796	430	680	572
Cercospora zeae-maydis	610	480	664	670	680	682	440	562	592	410	634	534
Chlamydia muridarum	24	26	24	62	14	28	30	72	66	34	56	100
Chlamydia trachomatis	4	6	4	24	32	28	20	10	30	28	32	14
Citrobacter rodentium	28452	23590	28040	21066	21054	24594	20224	28266	24304	19424	24464	22108

Clavibacter michiganensis	3706	3280	3870	3296	3480	4056	3028	3704	3928	2840	3716	3078
Claviceps purpurea	3664	2798	4282	3128	3442	3810	3940	4646	3590	3370	4132	4508
Clavispora lusitaniae	0	0	0	0	0	0	0	0	8	0	0	0
Clostridioides difficile	30924	23544	32096	24552	26060	29416	24658	32030	29272	22172	29052	25776
Clostridium perfringens	138	94	168	104	194	166	102	138	144	96	132	88
Coccidioides posadasii	706	990	828	690	888	722	736	744	610	408	620	608
Colletotrichum gloeosporioides	4668	3154	4754	4004	3676	4424	4776	5940	4950	4314	5096	5750
Colletotrichum graminicola	2354	2746	2526	2582	2576	2786	2746	3440	2692	2182	2650	3078
Colletotrichum higginsianum	402	284	428	420	372	420	580	640	490	428	474	626
Colletotrichum lagenaria	5902	5856	6220	6274	6818	6914	6698	8060	7010	4980	6464	6610
Colletotrichum lindemuthianum	2460	1780	2256	2590	2904	2474	3312	3526	3116	2382	3088	3322
Colletotrichum orbiculare	8094	6566	7412	6578	6584	7452	7148	9286	7424	6134	7944	8434
Colletotrichum trifolii	78	44	68	104	182	60	162	90	76	60	102	186
Corynebacterium diphtheriae	2652	2380	2728	2776	3012	3636	1750	2324	2036	1498	1900	1536
Coxiella burnetii	13950	10926	10698	7638	7986	8652	8352	11604	9098	7326	9532	9006
Cronobacter turicensis	4394	4490	4400	3918	4094	3924	4166	5346	4662	3886	5336	5464
Cronobacter universalis	166	240	180	240	210	206	220	308	200	180	228	208

Cryphonectria parasitica	2000	974	860	546	468	580	816	1032	870	574	1024	1062
Cryptococcus gattii vgiii	1266	1084	1378	950	920	1196	1108	1464	1236	958	1114	984
Cryptococcus neoformans	124434	108962	121960	108308	112050	116334	116422	144796	124722	96334	124816	127772
Dactylellina haptotyla	2	4	8	2	4	14	0	2	0	8	2	0
Dickeya dadantii	76498	71398	81494	68184	69202	73464	72650	95424	81642	65094	82610	84392
Dickeya solani	1600	1542	1766	1424	1326	1694	1366	1898	1716	1160	1598	1302
Edwardsiella ictaluri	32742	30598	31898	29044	28238	30326	28972	34686	30990	24942	31748	30766
Edwardsiella tarda	3506	2424	2676	2196	1948	2150	1800	2444	2320	1914	2476	2170
Enterococcus faecalis	23156	19158	22232	16442	16814	19324	17800	21640	19786	15714	20560	19360
Enterococcus faecium	17070	10840	16046	9220	9848	10480	12550	16796	12578	11838	13604	14380
Epichloe festucae	898	914	962	708	808	1112	742	1014	768	552	816	830
Erwinia amylovora	176130	162280	180020	145812	147574	160622	152234	201754	172544	137140	174900	173396
Escherichia coli	234042	208312	238618	185100	188860	210568	190794	272184	229338	174408	217404	218782
Exophiala dermatitidis	80	84	114	108	154	152	108	156	126	138	118	168
Exserohilum turcicum	1802	1654	1868	1918	1874	2252	1736	2074	2062	1728	2066	2044
Flavobacterium psychrophilum	84	106	84	196	264	132	204	206	184	154	120	186
Francisella tularensis	43474	37306	42726	37872	38504	40992	39742	50926	43710	34288	45170	47304

Fusarium asiaticum	500	464	536	644	576	634	796	766	832	644	1138	1502
Fusarium fujikuroi	652	506	536	640	632	720	622	746	600	594	492	592
Fusarium graminearum	276970	237262	275438	251036	265406	275540	280086	332702	289802	216506	286212	299414
Fusarium oxysporum	7214	5434	6888	5494	6322	6282	7950	8286	7584	5586	7048	7984
Fusarium proliferatum	2064	1244	2292	1298	1236	1302	1328	2082	1276	1288	1228	1604
Fusarium pseudograminearum	40	24	10	8	8	6	12	8	12	6	4	10
Fusarium sambucinum	36	12	30	32	48	42	144	134	80	24	26	40
Fusarium sporotrichioides	24	6	124	6	18	12	10	0	0	10	16	10
Fusarium verticillioides	22448	21032	23960	21488	21896	23984	20578	26728	23628	17966	23548	22680
Gaeumannomyces graminis	1800	858	920	518	470	542	398	408	522	388	700	368
Glaesserella parasuis	15796	14568	17162	15008	14958	16816	13382	18784	16720	13084	15946	15216
Gloeocercospora sorghi	214	204	154	342	334	298	306	298	302	180	240	286
Grosmannia clavigera	62	62	82	32	62	94	130	102	58	48	64	94
Haemophilus ducreyi	1224	1524	1494	1112	1004	1126	1106	1634	1230	978	1204	1142
Haemophilus influenzae	9470	7740	9164	7928	7486	8392	7416	9096	7584	6280	7774	7594
Helicobacter pylori	30208	27840	29480	25378	25850	28064	25926	34200	30660	22500	28168	27380
Histoplasma capsulatum	160	212	204	156	156	210	150	234	184	116	182	238

Hyaloperonospora arabidopsidis	550	314	450	430	422	482	558	662	596	568	656	742
Kingella kingae	7578	7232	8898	7678	8376	7984	7780	8992	7448	5962	7040	7744
Klebsiella pneumoniae	173202	158452	178160	144298	144926	163734	145870	194572	169430	132462	168866	162652
Lactococcus lactis	186	164	192	202	260	282	238	472	384	238	302	294
Legionella pneumophila	45878	38070	49900	36714	37984	41110	38276	51808	43518	36106	42906	41430
Leishmania infantum	3400	3244	4048	2756	2928	3034	3076	3680	3428	2710	2946	3196
Leishmania major	22764	19414	21548	18682	19606	21252	19660	25266	22422	16960	22416	21162
Leishmania mexicana	12612	12336	12920	11344	10762	11424	11780	15312	13090	10020	13006	13066
Leptosphaeria maculans	9488	8034	9128	7870	8680	9274	8356	10520	9194	6648	8934	8776
Leptospira interrogans	2378	2188	2718	2712	2600	2432	2344	2956	2576	1890	2632	2550
Listeria monocytogenes	89852	80248	90378	78972	81748	89922	85236	107386	93826	71068	95834	93846
Lonsdalea quercina	0	0	0	6	2	0	12	0	0	0	0	0
Magnaporthe oryzae	240744	195764	243422	204958	212258	234600	218562	270288	237432	180336	237286	231720
Metarhizium acridum	11482	8572	10870	8480	8230	8614	8902	12378	9670	7772	9860	9950
Metarhizium anisopliae	1146	1102	1354	1110	1234	980	1836	2278	1672	1440	1532	2128
Metarhizium robertsii	11312	11110	10894	10558	11494	12138	11538	14804	12068	9342	12138	11794
Monilinia fructicola	2	0	2	4	4	0	16	44	24	6	10	14

Mycobacterium avium	5452	4836	6428	4906	5206	5034	5728	7168	5948	4812	6054	6260
Mycobacterium marinum	5046	3252	4376	2322	2628	3412	2284	3542	2882	2370	3076	2016
Mycobacterium tuberculosis	200598	163654	198120	159758	158334	183294	150062	192408	174908	136384	178202	160592
Mycobacteroides abscessus	1632	760	1904	708	654	1206	612	1622	1382	690	888	474
Mycolicibacterium smegmatis	1364	920	1466	1084	1190	1500	1110	1124	1430	964	1550	820
Mycoplasma agalactiae	22562	16964	22176	17490	17538	19958	19638	24636	21592	16218	21504	22252
Mycoplasma gallisepticum	3026	2896	3484	3240	3468	3754	3164	3660	3454	2606	3544	3108
Nectria haematococca	200	146	174	252	170	172	160	160	224	110	214	236
Neisseria meningitidis	39868	38338	44214	36952	37052	42088	37098	51764	46098	34670	43690	43396
Paenibacillus larvae	21424	20422	25062	16666	18540	23146	15786	21066	21604	14188	20698	15756
Pantoea ananatis	29898	28660	31014	28306	28818	32060	29678	39270	34096	28566	36944	38650
Pantoea stewartii	10616	8020	9854	7658	7940	9596	8186	10784	10760	7960	9368	8796
Paracoccidioides brasiliensis	2	6	12	8	12	8	34	36	50	14	34	58
Parastagonospora nodorum	44392	39320	43346	35134	36060	40466	37624	49152	41546	32942	42246	41108
Passalora fulva	1254	1020	1284	1538	1216	1692	1632	1866	1606	1132	1880	1944
Pectobacterium atrosepticum	26220	22192	25230	20884	21362	23650	20964	30282	24522	18878	24034	22818
Pectobacterium carotovorum	7058	5786	6896	3842	3994	4818	4164	6678	5032	4196	5184	4550

Pectobacterium wasabiae	4416	3642	3720	3510	3390	3744	3356	4424	4128	3176	4072	4020
Penicillium digitatum	2964	1814	2100	1900	1774	2118	1888	1816	1756	1564	1894	1956
Penicillium expansum	5714	5344	6046	5608	6388	6070	5866	6780	6142	5120	6520	6668
Photorhabdus luminescens	462	302	508	602	564	618	416	472	472	358	448	418
Phytophthora capsici	0	74	0	0	0	0	0	0	4	0	0	0
Phytophthora infestans	1136	990	1350	1138	1360	1300	1848	1750	1846	1408	1502	1756
Phytophthora parasitica	48	30	12	6	22	12	30	20	10	8	12	8
Phytophthora sojae	5016	4066	4112	3644	4208	3958	5204	5858	5080	3976	4946	5484
Plasmodium berghei	32	18	18	50	88	22	122	148	58	48	66	138
Plasmodium falciparum	366	280	522	536	444	436	378	492	340	384	502	384
Porphyromonas gingivalis	46820	38238	46276	37298	39100	42924	39350	49816	43932	34514	43100	38680
Proteus mirabilis	3306	2836	2626	2426	2538	2744	2350	3016	2958	1930	2988	2000
Pseudocercospora fijiensis	4	6	14	18	28	14	36	58	52	18	18	18
Pseudomonas aeruginosa	414800	372774	419136	314198	324018	362660	324512	456564	388448	313710	378832	371922
Pseudomonas cichorii	46236	42218	50586	34488	41382	46564	32212	40680	37274	29584	39038	35722
Pseudomonas fluorescens	1572	1284	1378	928	800	878	1100	1524	1040	1048	1094	1100
Pseudomonas savastanoi	26208	20014	24886	22072	22518	27160	20090	24242	24088	18228	23560	20858

Pseudomonas syringae	115814	99006	117204	90698	91728	102858	93074	126294	109632	86586	107368	104594
Puccinia graminis	40	54	58	54	24	48	40	36	26	36	26	60
Puccinia striiformis	914	936	842	700	950	1050	642	1110	928	672	1040	848
Pyrenophora teres	4	12	4	18	10	0	4	2	8	0	12	0
Pyrenophora tritici-repentis	16	36	12	28	24	24	172	188	150	58	100	126
Radopholus similis	312	270	310	322	506	392	640	540	366	232	356	662
Ralstonia pickettii	1384	866	1404	850	918	786	706	886	932	704	856	1092
Ralstonia solanacearum	72952	58398	74430	60664	60934	71372	58064	75634	70380	53156	68312	62516
Rhizopus oryzae	14	30	18	44	36	18	98	106	52	36	76	84
Rhynchosporium commune	64	100	80	96	238	100	248	302	164	90	108	208
Riemerella anatipestifer	12788	9576	11198	9638	9956	10770	10602	13444	11526	8704	11090	10454
Rosellinia necatrix	54	22	16	60	32	52	22	44	18	38	44	30
Saccharomyces cerevisiae	8078	5632	7464	5904	5948	6188	7304	9616	7670	6552	7988	8480
Salmonella enterica	392614	354122	396118	325662	334512	371934	332258	440080	393296	297276	389372	368492
Sclerotinia sclerotiorum	18376	14602	18462	15696	15590	17982	14864	18954	16930	13466	17826	15570
Septoria lycopersici	4158	2190	3482	1328	1432	1630	1962	3134	1986	2138	1878	2352
Serratia marcescens	1824	1638	2016	2004	2016	1942	2220	2716	2300	2058	2506	2446

Shigella flexneri	152	142	242	40	102	222	60	638	790	100	22	10
Staphylococcus aureus	258570	217824	261956	215812	221512	248864	221574	289248	250842	195440	262498	247522
Stenotrophomonas maltophilia	518	376	512	408	416	402	420	680	546	398	512	514
Streptococcus agalactiae	340	440	396	484	452	618	316	452	384	286	458	336
Streptococcus parauberis	1236	1144	1306	1272	1206	1406	1772	1938	1684	1152	1930	2020
Streptococcus pneumoniae	121040	106768	129056	111292	114808	124470	116538	151248	131076	101074	131636	129056
Streptococcus pyogenes	30938	27194	31810	25936	26132	30282	26444	34026	29868	23036	30168	27458
Streptococcus suis	107890	102104	110892	101660	100714	112800	95104	119946	106482	82862	108636	104024
Streptomyces scabiei	520	494	486	442	414	528	324	558	546	338	642	342
Toxoplasma gondii	3890	3722	4398	4176	5208	4320	6948	7496	5786	3826	5476	6780
Trichoderma virens	204	244	140	244	208	66	224	78	96	28	80	100
Trichophyton mentagrophytes	2646	3154	2840	1638	2378	2384	1410	2200	1612	1248	1736	1496
Trichophyton rubrum	38	24	82	52	54	68	30	48	28	70	60	74
Trypanosoma brucei	124	66	146	194	202	206	198	230	252	172	222	142
Trypanosoma cruzi	10958	9030	9580	8026	8274	8890	8720	11882	9380	7496	9484	10542
Ustilaginoidea virens	522	446	636	790	796	720	698	798	652	428	756	806
Ustilago hordei	16	2	6	18	8	34	18	2	14	2	2	6

Ustilago maydis	11086	7432	10010	9114	10758	9474	13516	15060	12052	8226	10732	13082
Venturia inaequalis	6	16	20	10	10	16	12	26	30	16	28	34
Verticillium dahliae	24342	21714	25372	21608	22432	23478	23680	30266	26142	20956	25518	26744
Vibrio anguillarum	0	0	6	14	6	4	2	0	6	2	10	2
Vibrio campbellii	428	590	632	462	490	496	342	572	392	326	438	326
Vibrio cholerae	103820	109870	108664	93112	94184	101494	91784	126062	110864	86286	108984	111900
Vibrio harveyi	6384	7012	6608	6416	6152	6604	6510	8578	7328	6184	7640	7890
Vibrio parahaemolyticus	384	1076	528	464	472	466	432	756	554	440	572	568
Vibrio tasmaniensis	5572	7158	5836	5978	5386	6412	6016	7852	6660	4664	6534	5924
Vibrio vulnificus	9248	9580	10612	8692	8726	8568	8014	12824	9774	7584	8244	9486
Xanthomonas albilineans	5016	3108	5102	3680	3784	4074	4408	5718	4382	3852	4392	4692
Xanthomonas axonopodis	798	806	932	884	840	1154	708	982	986	682	806	640
Xanthomonas campestris	150568	131472	151306	122642	118224	131286	121030	166894	146530	117344	143248	139530
Xanthomonas citri	64678	49370	62854	35666	34682	39276	38652	59532	45782	39944	41822	42402
Xanthomonas hortorum	2540	2940	2350	2084	2206	2584	2402	2626	2272	1692	2124	2058
Xanthomonas oryzae	309976	265700	304896	235346	239698	265446	241842	327746	283602	226442	288392	271366
Xenorhabdus nematophila	6362	6514	7708	6104	6120	6924	6136	9136	7924	5960	7002	8010

Xylella fastidiosa	34752	27028	37130	21578	22162	24020	25478	41086	33092	26892	27532	30700
Yersinia enterocolitica	1682	1220	1482	756	672	754	832	1164	908	864	1014	1060
Yersinia pestis	42018	36544	41394	29920	29260	34328	33126	47796	39964	31838	38212	40568
Yersinia pseudotuberculosis	40876	35830	44282	34906	34572	38422	35426	47414	41314	31830	41878	40492
Yersinia ruckeri	1896	1856	2128	1884	2018	2374	1480	1892	1904	1132	1652	1272
Zymoseptoria tritici	24978	18440	22970	19614	20742	21236	21358	27578	22642	18564	23012	23802

Table S2 Abundance of VFGs annotated in the VFDB database

VFs	CK1	CK2	CK3	MA1	MA2	MA3	MO1	MO2	MO3	OA1	OA2	OA3
CVF254	12	0	0	2	8	6	2	2	6	0	2	0
VF0093	4	4	10	8	6	6	2	2	0	10	2	4
VF0261	6	6	4	2	8	6	14	12	0	4	6	2
VF0288	14	14	34	2	6	18	8	12	26	2	10	6
VF0406	4	124	12	2	2	0	0	2	6	2	0	0
CVF756	26	44	16	36	40	48	10	18	10	4	2	8
VF0093	10	24	18	32	22	32	20	12	14	34	24	22
VF0111	16	34	30	16	20	42	34	38	36	10	40	8
VF0298	62	20	60	6	66	26	28	22	34	36	50	24
CVF517	58	82	38	48	78	66	24	26	26	36	70	34
VF0190	10	20	18	60	76	38	66	136	84	60	58	74
VF0294	60	44	76	56	112	90	62	40	74	60	76	32
VF0317	36	148	84	80	52	54	72	74	60	30	106	36
CVF330	122	96	164	94	58	106	46	68	108	54	134	68
CVF421	112	88	68	92	108	104	166	204	158	126	112	90

CVF778	150	112	156	136	182	148	128	204	284	114	134	164
CVF252	252	218	254	130	140	118	114	168	174	106	146	106
VF0166	174	70	166	196	202	228	192	334	212	194	240	246
VF0260	174	538	202	250	304	284	238	188	272	148	184	210
CVF255	338	270	302	288	278	282	234	228	328	170	254	158
VF0297	356	286	376	272	328	382	152	230	224	184	214	126
VF0295	310	334	436	270	284	340	236	302	306	234	330	350
CVF663	428	258	390	290	372	534	286	288	346	236	432	192
CVF335	384	316	450	386	444	372	510	550	426	428	592	510
CVF627	346	348	328	322	472	448	468	720	484	370	532	598
VF0471	296	240	460	416	412	446	432	668	492	492	534	650
CVF365	408	844	564	526	526	488	460	590	544	544	524	590
VF0571	614	572	794	420	450	680	448	936	768	464	400	488
VF0300	894	790	790	824	834	960	530	432	708	474	600	396
CVF362	678	574	802	748	668	710	656	866	776	534	670	652
VF0405	894	530	792	724	702	762	694	1120	826	574	836	856
VF0262	824	712	1064	938	1078	974	1076	1442	1120	722	1086	1074

VF0336	1096	1418	1266	938	984	888	1032	1294	1274	758	1088	1082
VF0112	998	1324	1358	1128	1230	1340	1166	1692	1262	996	1256	1204
VF0113	1312	1438	1428	1462	1446	1500	1340	1936	1724	1298	1636	1678
CVF328	1832	1812	1838	1572	1700	2000	1556	2098	1808	1440	1996	1804
CVF253	2128	2126	2308	1854	1704	2226	1522	2178	2160	1554	1546	1296
VF0368	1784	1844	2272	1992	1968	2104	1862	2312	2188	1694	2028	2042
CVF010	2534	2882	3286	2054	1980	1986	2182	3446	2534	2350	2436	2664
CVF364	4048	2474	3752	2698	2560	2486	3052	4150	3628	2830	4172	4114
CVF667	4694	3524	3876	3652	3742	4256	3068	3690	3668	3000	4098	3242
CVF332	4124	3502	4596	3798	3854	3832	3328	4424	3814	2880	3548	3306
CVF387	3614	3336	3822	3698	3828	3930	3916	5236	4184	3436	4230	4590
CVF664	4564	4266	4466	4756	4942	4910	4566	5500	5244	4026	5400	5612
CVF329	6678	4326	6574	4608	4512	5190	4742	5852	5586	4912	5552	5340
CVF327	5496	4498	5456	5668	5920	6100	5764	6254	6016	4658	6194	6180
CVF331	8468	6066	7860	5322	5574	6510	5986	8208	6634	5652	6864	5918
CVF377	6272	6206	7808	5898	6730	6342	6946	9434	7604	6274	8098	7474
CVF666	7572	8000	8078	8028	7918	9266	7000	9724	8636	5986	8130	7282

CVF334	8642	8350	9644	7880	7784	10626	7162	8376	8704	5634	8304	5698
CVF333	9192	8482	9960	7250	7534	8474	7262	9700	8178	6656	8364	7544
CVF325	10088	8558	9806	8348	8272	9358	8212	10536	9018	6992	9772	8548
VF0287	12846	12760	13378	11382	11380	12000	11026	15410	13808	10388	13176	13300
VF0286	21794	17952	21504	16564	17056	20644	15118	20134	18588	13804	18118	15000
CVF529	24392	29142	27446	24048	25432	27012	25228	33674	27926	21470	28550	25668
VF0463	33508	32334	34276	27872	29754	33404	28568	40012	33888	25688	32674	30426
CVF331	39072	33968	37180	29566	30622	35876	29992	39048	35292	26290	34542	32432
SS120	0	0	2	2	0	0	0	0	0	0	0	0
CVF639	2	2	2	0	0	0	0	0	0	0	0	0
CVF867	20	0	2	0	0	0	0	4	0	2	0	0
VF0493	0	0	0	4	16	26	2	4	6	12	0	2
VF0116	22	16	52	16	12	36	52	4	28	24	62	2
CVF039	168	240	114	188	158	164	216	270	254	220	198	94
VF0325	324	146	162	204	222	140	224	304	258	116	178	262
CVF641	194	150	280	292	226	264	164	380	234	156	212	158
VF0116	304	148	322	206	256	342	170	710	724	100	168	58

VF0372	1478	526	324	240	196	214	226	256	236	206	326	308
VF0499	778	678	782	650	630	914	574	882	848	666	880	618
VF0026	824	852	1120	974	862	1006	904	1348	1052	756	1052	1070
VF0221	1080	796	1172	852	952	1078	936	1212	1232	686	1108	984
CVF799	1482	944	1532	694	774	944	936	1462	882	966	1046	1230
VF0428	6982	5014	6496	5490	6112	7334	5146	7422	7144	4944	6490	5504
VF0394	11644	12418	12838	9454	10758	12192	9458	13956	13040	9822	12010	10422
VF0132	0	0	0	0	0	0	0	0	2	0	0	2
VF0282	0	0	0	4	0	0	0	6	2	6	2	0
VF0036	4	4	10	2	0	8	6	10	2	10	4	4
VF0323	0	22	4	0	0	0	0	32	6	0	0	0
VF0031	20	0	38	38	6	10	2	38	42	8	30	2
AI111	80	64	118	128	66	126	126	238	156	198	202	148
AI113	216	262	194	212	130	254	186	174	210	130	172	144
CVF189	300	214	254	268	198	236	156	168	186	202	234	200
CVF189	1948	2208	1736	1754	1830	2164	1452	2072	1876	1646	1984	1626
VF0239	1412	2024	1712	1830	1770	1570	2048	2116	2010	1756	1986	2868

VF0236	4794	2918	3762	2992	3010	3510	3316	4622	3916	3204	3880	3692
AI111	8226	8556	8840	7828	7686	8864	7736	11356	9238	7240	8734	8862
VF0323	13236	12840	14330	13042	13506	14566	13304	17412	15076	11698	15386	15346
VF0348	0	2	0	0	0	0	0	0	0	0	0	0
VF0399	0	0	0	0	0	0	0	0	0	2	0	0
CVF754	2	0	0	0	0	0	0	0	0	0	0	0
CVF743	0	0	2	0	0	0	0	0	0	0	0	0
VF0514	0	0	2	0	0	0	0	0	0	0	2	0
VF0516	0	0	0	0	0	0	2	0	2	0	0	0
CVF750	0	2	0	0	0	0	0	2	0	0	0	0
CVF033	0	0	0	0	0	2	0	0	0	0	2	0
CVF103	0	0	0	0	0	0	2	0	0	0	4	0
AI075	0	0	2	0	0	0	2	0	2	0	0	0
VF0352	0	2	2	0	0	0	0	0	0	4	0	0
CVF788	0	0	0	0	2	0	2	0	0	0	0	4
CVF802	0	0	8	2	0	0	0	0	0	0	0	0
CVF076	0	0	0	0	0	0	0	4	0	0	0	6

CVF451	2	4	4	0	0	0	0	0	0	0	0	2
VF0097	0	0	0	0	0	0	0	14	0	0	0	0
VF0102	0	0	0	0	0	2	0	8	4	0	0	0
CVF241	0	0	0	0	8	0	0	0	0	2	4	0
CVF797	2	0	0	2	2	0	0	0	0	0	4	6
CVF047	2	0	0	0	0	8	4	2	0	0	0	0
CVF792	2	16	2	0	0	0	0	0	0	2	0	0
SS001	0	6	0	2	0	6	2	0	0	6	2	0
CVF744	8	2	0	0	2	4	0	4	2	2	0	0
CVF872	0	2	0	2	12	0	2	6	0	0	0	0
CVF749	0	4	0	2	0	6	0	0	8	4	2	0
VF0129	0	26	2	0	0	2	0	0	0	0	0	0
CVF487	0	2	0	0	0	6	8	6	2	4	2	0
CVF865	10	2	0	0	2	0	16	0	0	0	2	0
CVF167	4	0	2	4	2	0	12	8	0	0	0	0
VF0258	0	2	2	4	4	0	0	4	4	0	8	6
SS194	2	2	0	10	4	6	0	4	4	4	2	2

VF0544	2	0	10	2	6	8	0	2	2	0	0	10
VF0009	8	0	0	0	6	0	6	8	14	6	2	0
CVF128	32	4	6	4	4	2	0	0	0	0	0	0
VF0477	2	18	2	0	0	0	0	36	2	0	2	0
VF0224	6	6	4	6	8	10	0	14	2	4	4	0
AI102	6	0	6	6	8	2	2	10	14	6	0	4
CVF679	0	12	0	20	0	0	0	0	22	0	2	10
CVF462	0	54	14	0	0	0	0	0	0	0	0	0
VF0477	0	50	4	0	0	0	2	0	16	0	0	0
VF0426	8	12	10	4	8	0	2	8	2	12	8	8
CVF793	4	12	0	4	4	8	2	8	6	10	12	14
CVF352	8	4	16	2	4	10	4	24	6	6	6	10
CVF507	40	4	12	4	14	10	0	8	4	0	6	0
CVF367	0	2	2	4	8	14	2	10	2	12	46	6
SS119	42	8	24	4	2	6	0	2	2	2	10	8
CVF194	2	8	6	6	4	0	14	32	20	0	2	16
VF0060	32	40	6	2	0	8	2	6	6	2	6	2

VF0574	2	12	4	22	10	44	10	0	8	2	0	0
CVF782	8	24	14	4	8	0	2	32	8	6	6	2
CVF232	2	10	14	14	22	22	2	2	22	0	2	2
VF0506	10	6	6	0	4	2	8	16	12	12	16	26
VF0534	8	6	8	0	12	26	34	6	20	4	6	2
CVF130	16	10	4	28	14	30	8	4	6	4	2	6
VF0458	2	6	16	8	20	2	10	38	10	6	8	6
CVF748	8	6	14	4	4	40	2	18	26	4	10	6
CVF806	12	14	12	6	12	18	8	10	14	10	24	2
CVF603	2	8	2	2	14	8	8	16	10	20	42	12
VF0002	12	12	18	8	12	30	12	0	28	4	8	4
CVF368	10	0	18	12	2	10	24	24	8	12	16	18
CVF874	18	12	40	14	10	8	6	16	8	4	16	8
CVF041	6	24	10	28	4	12	10	20	12	4	24	8
VF0081	4	24	2	12	16	18	6	8	12	30	30	2
VF0509	58	6	30	2	4	22	8	10	6	6	10	4
VF0137	22	4	12	20	34	14	26	10	28	2	10	4

CVF599	40	22	40	0	2	6	6	20	10	4	36	4
CVF195	14	48	60	16	8	12	8	2	2	16	2	4
CVF227	18	10	6	38	40	22	16	10	10	4	8	16
VF0404	36	4	36	2	8	26	0	68	26	8	0	0
VF0017	12	12	12	16	14	12	46	2	26	10	24	30
CVF098	34	18	28	16	16	8	20	24	12	10	24	10
CVF741	10	22	24	40	38	42	4	2	12	8	14	16
SS038	10	20	18	16	12	34	18	38	22	10	34	6
CVF561	18	10	38	10	40	46	14	10	22	14	22	12
CVF430	16	28	30	44	28	14	14	10	38	14	18	6
CVF827	4	2	0	4	6	6	48	48	12	26	32	84
CVF113	4	6	4	40	76	28	16	22	26	20	22	14
CVF018	14	10	30	12	12	12	4	110	56	0	4	20
CVF509	16	6	4	28	30	52	48	14	58	12	12	8
CVF638	26	24	22	10	14	16	28	58	30	10	42	24
VF0505	20	6	54	22	16	34	12	50	32	8	40	18
CVF304	68	18	28	16	18	48	16	18	32	18	24	8

VF0222	10	6	24	18	16	26	36	28	42	40	38	30
CVF131	10	0	2	28	22	32	56	16	62	34	40	20
AI317	14	8	10	26	16	30	60	38	70	20	34	8
CVF049	56	2	22	18	4	28	30	72	26	12	54	18
SS211	30	28	38	66	58	56	10	8	12	6	22	16
VF0064	18	24	28	28	36	22	26	56	32	42	22	32
CVF048	60	24	68	20	40	12	14	14	50	14	38	14
VF0578	18	8	10	14	28	40	30	34	82	54	22	38
VF0035	22	8	48	10	18	56	4	74	102	8	16	14
CVF794	12	84	20	42	24	16	20	60	14	16	34	38
CVF113	58	14	30	14	62	52	34	6	64	24	30	4
CVF684	44	12	50	4	20	38	12	106	108	0	0	0
CVF257	34	22	42	36	18	42	48	48	30	20	38	20
CVF610	80	26	36	32	22	30	32	28	48	30	36	10
CVF782	46	14	22	24	22	52	50	32	38	28	58	36
VF0275	54	16	48	42	44	22	14	24	28	36	92	16
VF0371	44	42	52	12	32	20	14	110	64	8	14	32

CVF425	24	22	32	40	38	32	26	76	86	12	32	30
VF0577	8	2	30	12	14	36	80	138	60	18	32	26
CVF265	34	42	120	64	44	74	18	26	26	14	14	10
VF0149	56	14	42	38	16	38	36	102	46	42	42	24
CVF822	30	48	42	50	64	46	40	30	30	36	46	38
VF0105	28	32	68	30	32	72	12	74	50	16	66	28
CVF510	68	22	20	18	18	34	48	56	94	84	42	20
CVF425	44	26	48	22	22	60	18	106	142	14	10	20
CVF486	44	76	66	36	40	52	48	46	40	30	28	32
CVF347	10	20	14	10	66	42	70	136	44	46	58	26
VF0527	50	54	32	28	70	92	58	38	52	16	22	32
CVF402	32	26	56	38	38	52	72	90	30	38	46	42
CVF015	42	74	38	30	50	36	66	56	34	34	46	62
CVF789	88	34	100	40	58	20	30	30	40	58	58	20
SS054	56	56	66	70	46	32	60	66	48	24	32	42
SS156	64	16	40	32	14	44	54	138	88	30	36	46
VF0401	34	48	84	50	42	66	40	92	54	24	76	8

VF0102	54	68	54	42	58	90	42	30	60	40	30	68
CVF045	26	76	34	10	22	18	106	84	66	54	56	96
VF0220	44	62	80	46	32	80	30	78	68	24	108	36
CVF351	12	140	50	40	36	60	64	98	26	38	96	66
VF0525	60	16	38	54	52	16	160	122	162	62	30	20
VF0313	50	30	94	52	70	124	44	102	72	38	98	40
CVF099	86	86	86	78	52	120	66	66	48	44	70	28
CVF825	64	62	68	64	44	56	60	92	90	70	76	108
CVF231	182	126	136	70	58	72	36	34	28	22	42	52
VF0486	72	38	78	30	30	86	16	242	246	36	0	0
CVF796	80	38	112	40	40	132	28	168	138	22	62	26
CVF864	70	36	36	20	90	50	90	118	152	98	62	80
VF0174	36	292	112	24	40	68	58	72	58	26	94	42
VF0134	90	44	128	92	56	106	76	52	72	70	132	30
VF0237	80	102	70	120	94	66	68	50	82	50	128	84
VF0070	76	54	72	114	124	106	46	88	38	58	100	122
VF0507	58	56	94	18	34	86	32	226	322	44	18	12

CVF102	16	88	20	118	40	84	146	136	96	92	80	84
CVF785	28	76	62	52	68	50	60	112	122	102	132	146
CVF739	166	82	60	70	112	134	64	70	144	26	50	46
CVF229	46	52	64	94	80	50	98	174	112	84	74	100
VF0092	100	94	146	54	64	92	68	96	118	52	128	48
SS002	72	58	60	102	166	142	62	60	80	80	106	124
CVF049	78	100	120	116	102	216	66	94	76	50	56	46
VF0098	120	72	148	110	82	114	94	84	82	78	112	44
CVF805	64	22	54	66	204	66	198	174	78	54	32	146
CVF669	52	38	60	66	140	146	98	116	136	112	172	72
SS001	106	70	86	66	154	140	64	124	86	106	178	50
VF0121	76	70	126	22	28	134	20	328	386	52	2	0
CVF122	56	54	70	86	60	78	178	124	116	84	116	232
CVF757	134	260	62	74	74	144	58	146	110	78	72	60
VF0194	86	102	180	24	56	114	54	274	192	62	74	60
CVF644	130	52	76	68	102	98	88	110	162	124	124	152
VF0078	106	60	140	178	104	162	94	106	102	100	78	86

CVF784	64	94	222	68	90	84	70	158	148	88	82	158
CVF114	186	112	140	88	78	76	114	106	110	98	98	128
SS032	126	80	132	136	98	106	150	230	104	92	98	70
SS181	100	100	192	88	86	120	104	140	192	86	138	122
VF0283	108	80	98	70	140	150	110	200	144	100	170	130
VF0320	128	86	128	248	198	232	46	50	64	68	130	136
CVF233	88	162	132	164	128	168	106	164	114	108	150	88
VF0107	86	134	110	154	150	160	156	148	168	120	88	150
VF0145	110	42	142	102	142	146	192	186	140	174	130	202
VF0004	84	52	134	158	120	168	196	106	150	100	206	238
VF0397	132	84	120	44	66	170	38	450	528	72	2	6
SS052	184	164	266	132	110	182	150	96	116	122	102	102
SS183	100	290	150	150	118	158	140	140	100	92	176	122
CVF798	138	100	166	122	168	158	190	222	164	132	104	154
VF0142	138	196	176	176	202	192	196	122	198	72	120	92
VF0243	164	148	168	148	134	122	138	220	216	136	164	162
CVF492	146	354	168	160	156	204	118	128	180	102	164	90

CVF747	124	218	216	92	100	220	126	232	252	98	224	80
CVF403	190	134	236	242	216	166	148	120	188	96	190	102
VF0480	106	324	134	178	142	174	148	270	122	132	144	190
CVF634	162	162	270	132	130	162	136	260	246	146	152	140
SS018	182	280	158	238	192	210	104	182	142	146	184	106
CVF826	268	172	206	152	130	190	186	220	196	118	156	182
VF0265	244	196	244	122	240	252	182	234	156	118	160	150
VF0365	210	130	250	130	138	266	94	310	380	86	150	166
VF0445	114	222	138	300	218	152	226	286	290	144	216	138
SS125	172	144	268	98	120	184	70	524	556	116	130	78
CVF797	1002	346	238	30	120	58	114	104	88	80	172	140
CVF258	222	204	168	260	264	252	194	330	192	164	152	154
VF0446	158	172	162	246	238	242	170	366	216	226	216	150
SS053	148	122	128	92	100	120	362	304	276	186	282	456
CVF466	454	148	466	122	124	146	170	304	138	244	124	150
CVF354	174	152	120	164	228	158	248	228	218	270	344	324
VF0530	318	394	242	278	330	318	156	130	210	94	116	116

VF0575	128	210	190	180	320	196	200	248	254	240	252	318
CVF230	156	144	174	162	180	306	274	320	244	220	330	232
CVF740	204	188	202	244	196	294	140	396	310	312	310	172
CVF600	166	114	224	142	202	252	246	492	300	250	282	312
CVF792	278	472	282	250	162	298	224	284	218	224	206	156
SS034	194	162	182	134	138	184	334	426	242	330	356	532
VF0103	214	740	376	238	282	232	222	202	258	144	210	202
CVF346	314	218	302	218	308	346	242	272	342	238	254	266
CVF621	290	306	330	212	222	236	224	394	236	234	374	266
CVF847	150	194	158	188	140	224	322	364	344	278	498	470
CVF414	304	352	262	204	182	196	304	336	288	260	316	352
VF0576	352	174	266	286	324	326	204	244	316	276	286	314
VF0354	250	286	246	296	312	388	316	332	274	234	348	226
CVF853	170	194	232	310	276	274	376	436	330	236	312	446
CVF780	210	432	424	192	220	306	218	414	318	278	360	400
CVF804	238	176	356	360	284	416	320	436	314	250	320	312
VF0409	192	188	150	212	206	228	378	422	470	470	468	474

VF0349	278	284	330	278	290	378	366	448	348	306	378	280
VF0038	296	426	314	622	454	432	190	362	248	176	262	184
CVF388	262	190	306	248	260	314	282	410	480	350	470	412
VF0068	370	384	326	358	336	372	290	406	326	294	368	226
CVF112	128	82	146	162	168	190	522	622	450	460	458	720
CVF736	472	346	366	390	400	356	312	332	370	236	282	264
SS039	342	444	386	384	422	434	390	338	352	254	298	224
CVF228	252	250	232	326	362	302	448	612	406	336	362	484
VF0025	338	256	330	118	206	336	162	1026	1356	166	96	32
CVF560	338	312	292	332	412	448	368	426	416	258	398	450
SS208	388	378	418	302	376	366	500	426	422	268	332	322
VF0582	332	278	458	130	152	432	210	1022	1202	222	92	80
CVF635	270	294	326	366	454	430	402	536	362	340	444	498
CVF532	446	382	542	424	356	368	388	390	416	298	462	410
CVF624	102	68	62	74	116	32	728	584	452	384	784	1578
CVF404	362	566	348	410	464	448	488	438	478	274	428	332
CVF372	572	590	548	332	346	244	452	380	402	336	552	472

CVF263	372	470	478	530	442	600	402	542	460	372	436	488
CVF053	1396	428	1548	44	94	44	302	878	286	544	92	216
VF0513	554	740	806	208	262	590	208	950	944	200	384	184
VF0278	468	372	492	350	442	470	514	770	630	546	564	636
VF0503	426	552	560	460	454	558	456	844	556	454	600	464
VF0259	460	328	502	536	564	438	588	828	510	370	528	746
VF0402	504	344	524	450	460	490	552	606	620	584	756	718
VF0327	484	426	516	570	596	630	486	678	652	476	642	456
CVF676	510	448	478	614	522	628	494	736	540	464	610	634
VF0126	478	1258	744	446	470	376	430	812	536	354	638	582
VF0434	574	566	706	836	742	1140	416	888	522	356	578	326
CVF734	338	842	604	762	718	720	582	608	628	436	692	724
VF0369	678	678	820	616	630	634	512	794	746	504	772	828
CVF550	482	336	506	522	442	420	864	1226	638	446	976	1472
VF0140	658	488	632	584	802	832	740	840	858	606	802	814
VF0447	748	504	802	670	894	842	878	688	936	576	800	786
SS009	676	810	716	878	836	762	760	974	674	544	710	806

CVF106	564	524	640	682	600	516	820	1022	968	894	1028	1266
VF0431	836	766	938	786	848	972	730	1094	994	746	906	552
VF0346	712	552	772	732	1058	1032	778	1104	880	724	894	990
VF0118	884	1384	1030	970	884	920	688	1082	988	366	512	574
VF0398	794	1016	1424	784	558	932	750	1160	1280	504	994	632
VF0409	996	920	1008	754	942	1084	830	1122	938	624	880	852
CVF268	898	1384	792	870	878	858	816	1338	1038	892	1036	946
CVF453	902	948	892	866	1104	1118	836	1164	964	840	1008	1108
CVF035	1200	1234	1086	912	988	1128	776	962	876	730	1110	750
VF0566	1146	1272	1294	924	964	1066	888	1114	1030	598	870	778
AI345	500	410	438	426	448	402	1546	1550	1154	920	1504	3106
VF0041	2016	890	1506	798	756	800	900	1356	968	818	988	794
CVF861	804	1214	856	978	904	1066	1106	1312	1030	880	1370	1212
CVF389	764	840	946	1040	1018	1316	974	1756	1232	798	1088	1124
CVF353	1824	690	2066	580	682	584	860	1640	970	1250	930	844
VF0083	1028	1000	1176	936	1042	1368	910	1684	1114	668	960	1062
CVF862	894	1492	1064	1016	870	1096	986	1492	1024	798	1228	1082

CVF793	956	1008	1114	1020	1028	1156	1160	1288	1388	926	1002	1042
VF0458	894	862	1150	988	1066	1006	1108	1612	1248	866	1188	1152
VF0479	1292	830	1402	788	894	904	1018	1856	1110	1078	904	1206
VF0435	2530	1608	1274	1024	990	1412	602	1160	842	460	900	728
CVF643	1708	1144	1670	824	908	960	978	1352	1038	1102	934	968
CVF642	1302	1348	1298	1106	1272	1362	1054	1256	1238	720	1032	844
VF0410	988	1190	1222	1238	1100	1046	1098	1460	1344	1002	1250	1352
CVF298	1092	1094	1538	1190	1130	1650	912	1498	1474	676	1362	988
VF0191	1104	1366	1284	990	1120	1280	1052	1492	1562	1038	1506	1084
VF0156	1384	1270	1266	1258	1446	1440	1148	1568	1350	1028	1096	1126
VF0237	1284	1106	1366	1644	1442	1778	1112	1340	1382	1024	1116	1284
CVF032	1604	1468	1220	1316	1390	1484	1260	1668	1304	1116	1328	1494
VF0462	2418	3982	3234	410	712	600	954	1680	752	1238	618	524
CVF031	1508	1280	1612	1268	1406	1534	1328	1826	1380	1280	1606	1646
VF0069	1488	1250	1448	1472	1700	1400	1702	1492	1774	1146	1556	1294
VF0154	1570	1496	1492	1544	1508	1502	1304	1688	1564	1206	1832	1476
VF0581	1444	1346	1580	1480	1680	1874	1590	1632	2056	1372	2032	1144

CVF312	2588	1436	1806	1654	1408	1522	1340	1560	1480	1336	2136	1268
CVF783	1244	2412	1318	1268	1246	1266	1454	1936	1790	1632	1932	2144
VF0403	1968	2096	2036	1688	1752	2090	1398	1526	1400	1080	1410	1350
VF0127	1134	7494	1964	952	1614	1048	678	1602	746	880	1030	1354
CVF259	1352	1418	1366	1130	1358	1348	1634	2246	2270	1978	2102	2322
SS194	1760	1946	1994	1620	1844	2114	1430	1910	1796	1230	1860	1556
VF0521	1274	1286	1458	1470	1614	1676	2364	2532	2146	1306	1976	2374
CVF355	1594	1478	2076	2002	1866	2194	1572	2016	1748	1354	1980	1964
CVF636	1508	1972	1636	1540	1716	1962	1670	2450	1960	1640	1934	1948
VF0408	1698	1860	1912	1586	1636	1928	1756	2966	2144	1500	1998	1632
CVF116	1932	2958	1718	2386	1806	1734	1458	2352	1412	1288	1572	2282
CVF187	1710	1176	2038	1482	1818	2036	2100	2222	1878	1782	2368	2370
SS015	1452	1232	1420	1720	1820	1562	2140	2180	1996	1432	2864	3224
CVF547	3094	1810	2188	1604	1862	2008	1758	1908	1804	1294	2024	1764
CVF737	1770	1726	2080	1850	1850	2008	1818	2470	2168	1524	2010	1946
VF0512	1696	2660	1804	1710	1908	1768	1788	2540	1888	1468	1972	2082
CVF429	4918	2442	2968	1110	1064	1264	1322	2288	1528	1734	1778	1458

VF0321	2842	1812	2350	1882	1918	2080	1654	2366	2058	1348	2078	1714
CVF348	2028	1678	2298	2266	2266	2494	1942	2384	2220	1782	2078	1910
CVF548	2016	1634	2342	1960	2008	2202	2206	2176	2182	1704	2270	2858
VF0569	2370	2802	2988	1782	1894	1952	1928	2556	2058	1776	1912	1994
SS193	2166	1962	2690	1868	1900	2702	1946	3168	3194	1770	2334	1998
CVF171	2134	1820	2490	2318	2526	2852	2164	2822	2784	1950	2604	2350
SS177	2396	3282	2088	1284	1730	1616	2182	6312	2294	1630	1852	3038
VF0315	3706	2072	2542	1968	2294	2668	2164	2912	2522	2020	2780	2500
VF0154	2620	2408	2936	2632	2542	2870	2344	3500	2972	2226	2848	2484
VF0335	2708	2964	3168	2150	2158	2382	2436	3576	2448	2390	2858	3290
VF0035	2492	2216	3148	2250	2322	3702	1910	4210	4386	1936	2350	1944
VF0437	2946	2648	2954	2048	1972	2022	3110	3608	3042	2184	2786	4386
VF0488	5990	3378	3164	1998	1874	2148	2214	3198	2254	1968	2900	2860
CVF293	3426	2554	3458	3156	3190	3940	2502	2520	2764	2314	2942	2230
CVF046	2464	2772	2756	2710	2854	3112	2948	3536	3460	2382	3126	3200
VF0014	2640	2474	2906	2786	2676	3328	3260	3564	3554	2742	3456	3436
CVF411	2656	2648	3126	2456	3136	3636	2954	4060	4570	2452	2728	2730

SS038	4710	3346	3696	2862	3036	4000	2246	3984	3590	2236	2970	2802
CVF129	3366	2822	3762	2686	2870	3356	3060	4136	3462	2990	3630	3622
VF0579	3438	4374	3592	1520	1604	1722	2914	9812	3558	2288	2034	3440
VF0487	4966	2610	4328	2658	2964	2596	3126	4702	3208	2812	3120	4060
SS004	3800	3046	3704	2966	3336	3750	3408	4100	4094	3136	3774	3478
CVF519	3588	3676	4296	3500	3770	4216	3398	4610	4046	3276	3936	3822
VF0155	3294	3004	3626	3614	3508	4182	3592	4870	4536	3798	4836	4314
VF0019	4904	4872	4694	4234	5244	6650	3560	4778	3914	2304	3154	2508
CVF735	6912	3708	8160	2364	3110	2950	3812	5084	3792	4420	3620	3624
CVF545	4424	3768	4644	4260	4258	4480	4034	5210	4740	3800	4528	4768
VF0515	4158	3910	4084	3550	3908	3946	4124	5446	5132	4614	5194	5230
VF0333	4796	3584	4674	3274	3352	3618	4464	5864	4600	3976	5406	6120
VF0475	3960	4518	4380	3980	3974	4440	4068	5888	4748	4034	4592	5496
VF0429	5044	4556	5558	4286	4682	5494	4234	4800	4876	3462	4534	3570
VF0444	4406	4736	4692	4502	4566	5370	4804	5498	4796	3982	5180	5058
VF0326	4726	4752	5170	4822	4804	5784	4950	6372	5966	4408	5590	5048
CVF601	4300	4674	4758	4358	4460	4716	5160	7384	5828	4438	5704	7054

SS008	5836	4312	6710	4520	5132	5382	6138	7732	6390	4526	5504	5916
CVF465	6208	5296	7134	4846	5728	6360	4762	6412	5554	4930	6680	5198
CVF803	5516	4428	5750	4958	5514	5202	6676	7512	6696	5088	6162	7480
VF0075	5076	4926	5672	5050	5062	5682	6040	8038	6702	5364	6952	7638
VF0238	6516	5728	5464	5884	6062	6898	5530	7074	6508	4940	6692	4936
CVF785	6056	5832	6428	5578	5662	6132	4988	7604	6190	5970	6810	6474
VF0114	6540	5324	6568	6184	6142	6940	5860	7674	6618	5118	7290	6294
CVF417	7580	5390	7730	5686	5820	6720	6160	7576	6746	5058	7072	6702
SS003	6908	6362	7054	5740	5418	6128	5888	7614	7234	5822	6986	7162
CVF786	8106	5960	6682	5832	5650	6656	5758	8108	7288	5486	6944	7052
VF0476	10928	5748	9240	5790	5496	6870	5330	8344	6572	5106	6172	5810
VF0056	8976	6626	8574	5862	5614	6114	5742	8312	6656	5862	6688	6890
CVF780	5928	7868	6224	6522	6536	6294	6326	8534	6812	5470	8032	7694
CVF549	6918	5000	7484	6944	7038	7126	7492	8622	8230	6188	6872	7696
CVF123	8254	7482	8012	7758	7266	7760	7368	9834	8058	6474	8624	8586
VF0156	7278	7414	8270	6824	7044	7886	8334	11002	9362	7228	8824	9186
VF0322	7844	7382	8998	8088	7512	8032	8486	11034	9046	7430	8512	9904

VF0478	6666	7112	7180	7902	7448	7824	8110	11730	9758	8308	10358	12004
CVF546	9880	7516	9964	7780	8026	9696	8766	10566	9918	7840	9932	8946
CVF518	8228	9372	9056	8218	8378	9498	9012	12288	11110	9232	11226	11708
CVF521	11396	9200	10288	8788	8600	9920	8708	12224	10348	8574	10724	10402
CVF783	9496	9988	10058	8602	9098	10070	9620	12492	10792	8974	11666	11774
CVF784	13730	9028	11080	9592	9920	11018	9346	12584	10548	8048	11012	10084
CVF534	10822	9236	10536	10062	10124	10180	10412	14510	11854	9454	11516	13070
VF0356	10980	9446	11660	10580	10444	11840	10472	13598	11966	9200	11682	11774
AI097	11656	11886	11934	10174	9864	10678	10654	14454	12340	10158	13390	13738
CVF263	13348	13288	14878	14250	14492	14620	14150	17444	15408	11898	15808	14888
VF0460	14466	12886	15686	13488	14282	14914	14106	17796	15486	12240	16482	16300
SS026	21190	11154	23524	10206	10456	10672	13744	20932	13990	13954	14274	16690
VF0344	16068	16468	16070	16072	15478	16928	13572	17824	15266	11898	16458	14968
VF0207	14882	13050	15424	16212	16464	17930	14844	19674	15664	11972	16246	15742
VF0084	16600	15016	17664	14788	14898	15294	15730	21926	18568	15600	19332	20808
CVF299	17212	15198	16232	15128	15906	15970	17290	23036	18590	14378	18744	19966
CVF535	19126	20502	17646	17954	17372	19170	16380	20962	18240	14010	19372	17330

VF0159	19362	16200	19656	17482	17732	19318	16944	20978	18806	14580	20166	17096
SS047	19914	19310	20282	18196	18914	21356	19570	23890	20352	15672	20292	19276
CVF619	21766	20364	19844	18938	20842	21236	19488	24584	20884	16546	20880	21960
CVF803	22302	18658	21842	18678	18958	20408	19964	26506	22614	16896	21976	21964
CVF520	25642	18816	25104	20830	20686	23202	20140	25554	22838	17970	24110	21490
CVF521	28510	20940	28194	18380	20164	22102	21322	30260	25788	21922	25654	24802
VF0225	28736	23258	30256	24350	26504	28816	27290	35052	29570	23230	29812	30986
VF0430	40830	30742	42336	24284	26650	31158	27774	43942	37794	30394	33578	30542
VF0028	46584	40182	50542	41512	43938	46510	43290	57768	47644	37120	46782	46722
VF0273	57778	45854	58370	43108	42018	46720	45386	64964	54162	47128	55550	53342
CVF171	60792	49136	62112	52866	54608	62658	52396	63994	59772	46090	60004	56140
VF0473	73148	57800	75276	59022	58252	68408	55890	74190	66732	52444	65666	59274
VF0573	80232	64020	79228	65774	67106	74672	62172	77022	73036	55838	72574	67386
CVF786	83868	71868	86116	65120	64938	74064	64546	86408	74766	59872	76066	69216
VF0082	99694	94374	104688	84076	83200	92438	82566	117904	99974	82210	98486	96700
VF0334	123688	117016	123134	109490	112482	122292	102838	127260	114696	92868	119188	115188
VF0023	8	2	4	10	18	36	20	28	14	8	28	12

VF0048	64	210	158	40	58	66	28	46	56	10	12	24
VF0049	372	522	378	306	252	368	452	666	588	414	396	472
VF0024	550	710	588	456	512	496	460	772	450	386	394	420
CVF361	0	0	4	0	0	0	0	0	0	0	0	0
VF0061	2	4	4	0	0	2	4	2	0	6	2	0
VF0013	2	2	0	4	6	0	0	2	2	0	2	8
CVF460	6	2	0	4	0	16	2	4	0	0	0	4
CVF502	0	34	0	2	0	0	2	0	0	0	2	2
VF0012	12	0	8	0	2	12	4	4	6	4	2	0
CVF457	4	34	0	2	4	0	2	0	8	2	2	0
VF0357	10	0	12	6	0	6	4	4	12	0	6	0
CVF154	4	0	4	8	2	6	0	2	0	10	20	10
CVF091	10	4	8	2	12	18	10	4	10	8	4	0
CVF091	2	0	4	8	26	24	16	16	0	4	16	4
CVF504	0	12	6	8	16	2	20	16	26	2	8	12
CVF183	4	132	16	2	4	2	4	0	0	2	0	0
VF0267	18	48	6	6	2	4	10	22	10	12	24	10

VF0246	2	12	10	30	22	20	18	16	14	20	10	20
VF0249	6	16	20	0	4	0	20	94	8	22	4	26
CVF415	24	6	28	24	32	8	16	46	20	10	16	22
IA016	6	22	8	32	18	28	18	30	24	10	28	44
CVF051	10	18	20	40	28	22	28	10	38	14	42	22
CVF852	34	92	22	2	2	4	22	40	40	10	28	4
CVF835	46	34	40	24	36	22	22	14	46	10	36	24
VF0271	16	92	80	34	26	20	8	26	10	10	34	16
VF0047	14	84	20	28	32	16	26	114	22	10	14	6
CVF361	30	18	14	18	30	24	72	44	88	20	18	26
VF0046	44	106	76	16	8	44	24	18	20	22	22	4
IA014	72	46	52	18	32	52	28	18	34	10	38	22
VF0358	74	28	28	28	72	54	58	28	48	32	26	40
CVF201	62	14	34	34	54	42	114	82	48	80	84	88
VF0063	18	52	38	38	116	44	106	156	88	40	62	82
CVF184	50	138	90	38	32	106	40	64	84	42	128	30
CVF314	122	72	124	132	62	112	66	60	112	42	48	36

VF0390	132	84	68	94	116	162	92	72	82	24	52	68
CVF554	90	104	156	112	118	130	46	80	50	40	86	70
VF0268	206	92	170	66	76	72	72	112	108	102	124	148
VF0228	130	158	136	130	102	86	104	148	100	84	140	154
CVF359	150	62	164	160	212	200	74	88	118	84	118	110
VF0065	210	92	120	84	94	148	90	224	240	128	128	58
CVF276	182	170	202	116	136	140	204	266	156	142	120	126
CVF836	206	178	296	160	198	174	112	162	182	98	126	118
CVF768	204	230	242	298	260	242	292	312	260	162	354	224
CVF850	258	688	304	210	364	246	156	202	180	154	198	220
VF0551	266	270	414	228	182	370	244	440	314	184	272	192
CVF275	388	436	368	240	224	322	148	314	326	180	312	154
CVF456	224	240	246	354	338	348	310	414	310	246	310	352
VF0015	354	720	376	234	180	230	248	320	360	220	312	216
VF0270	136	324	110	124	176	122	722	510	424	242	400	646
VF0391	414	476	324	296	274	332	376	434	408	192	330	238
VF0389	788	228	872	176	130	68	318	628	332	492	186	150

VF0229	246	410	228	112	230	156	302	500	484	538	526	650
IA021	364	406	350	362	396	432	376	478	374	320	454	392
CVF851	492	384	552	406	394	524	406	458	586	400	358	446
CVF555	612	500	554	458	508	622	406	540	600	440	470	466
VF0293	2042	818	1100	310	240	348	292	550	328	460	404	308
VF0565	428	1192	676	444	492	426	440	760	718	582	588	746
VF0123	1290	612	534	182	204	222	756	728	676	426	816	1286
VF0289	748	670	984	598	590	636	546	778	684	538	562	496
VF0388	714	738	808	654	938	724	728	920	586	370	500	374
CVF478	422	760	516	400	460	470	716	1056	1088	946	978	1368
VF0291	1394	1082	1550	544	430	622	570	900	658	618	430	424
CVF461	1280	752	1716	522	514	568	738	1246	852	930	738	728
CVF477	1102	1042	1296	798	750	912	828	962	792	808	834	644
CVF513	1086	948	1160	994	908	1028	970	1160	922	794	1192	856
CVF479	1152	962	1106	1044	902	1038	1092	1208	1044	814	1142	1012
CVF849	1138	990	1186	1134	1158	1454	1044	972	1194	846	1148	992
CVF358	1514	1150	1692	858	926	1038	860	1476	1064	976	924	984

CVF316	1234	1232	1216	1280	1734	1396	1378	1520	1214	958	1336	1250
VF0269	1718	1564	1686	1474	1360	1692	1232	1936	1660	1452	1486	1554
CVF553	2220	1720	2360	1554	1784	1942	1754	2594	2120	1448	1896	1484
CVF556	1700	2300	1858	1992	1742	1840	1570	2450	2116	1664	1990	2206
CVF514	1854	1866	2054	1952	2118	2682	1832	2492	2326	1536	2450	1936
CVF197	2306	2006	2292	1978	2128	2418	1932	2218	2364	1754	2390	1914
CVF515	2480	2176	2828	1630	1898	2014	1914	2798	2170	1718	2088	2014
CVF552	2992	3788	3242	966	1120	1362	1550	2794	2160	1590	2102	2310
IA032	2060	1936	2132	2194	2216	2224	1998	2854	2448	1702	2484	2040
CVF278	2210	2522	2212	2194	2200	2138	2038	2788	2506	1950	1912	2628
CVF769	3616	2890	4610	1326	1544	1988	1430	3674	2892	1858	1490	1212
VF0550	2588	1988	3002	2516	2058	1998	2956	3748	3134	2604	3260	3876
IA001	4050	2798	3514	2074	2228	2694	2386	3598	3146	2314	2826	3066
IA005	4908	3630	4540	2434	2562	2404	2454	3498	2864	2778	2614	3282
CVF459	2732	2762	3052	2866	2968	3252	3276	4132	3232	2420	3498	4258
CVF274	2914	2914	3120	2952	3232	3380	3484	4042	3518	2948	3394	4156
VF0230	2806	3264	3528	2128	2436	2558	3252	4590	4102	3094	3860	4812

VF0292	3906	3274	3550	3262	2850	3252	3102	4236	3606	2896	3502	3636
VF0160	4334	3526	3424	3084	3030	3476	3540	4542	4070	2998	4304	4570
CVF668	4044	3598	4944	3878	4160	5492	3600	3446	4178	2766	4530	2582
CVF516	4018	4262	5118	3366	3446	4684	3262	4620	4494	2726	4712	3584
CVF386	7052	4198	6838	5264	4922	5352	5550	6392	5920	4786	6244	5956
CVF277	10566	6462	8694	3620	3174	3328	4898	8916	5746	5708	5196	6926
VF0136	6996	5474	7038	5366	5242	6354	5372	7212	7846	5148	7052	6254
CVF202	10574	7742	9090	6386	5886	7270	6394	8958	7784	6344	7758	7834
VF0256	11398	7506	10500	6496	6974	7520	7698	11306	10254	6880	8540	8456
VF0563	10034	9576	10786	9964	9852	11214	9512	12024	11774	7948	11006	9406
CVF501	10182	9952	10724	10112	10458	11032	9256	12230	10300	8210	11044	10504
CVF456	11554	9690	12534	9344	10136	11202	9448	11846	11594	8492	11666	9312
VF0299	16240	13060	16564	14140	14684	16416	13876	17172	16704	12156	15752	14352
VF0227	19248	15298	17724	13022	12930	14674	13164	17580	16034	12390	15670	14494
CVF358	18904	16006	18146	14414	13452	15660	13876	19288	16848	13418	16014	16116
VF0106	16266	12932	17816	13770	15008	16636	15770	19118	18056	13624	17528	17010
IA031	17944	15082	18372	15736	16130	18140	15936	20078	18374	12974	18756	17998

VF0228	18526	18870	21548	16466	17184	19688	16740	21928	19712	14400	20056	17694
CVF551	20278	16998	20740	18306	18842	22120	16786	21336	20592	15530	21058	18596
VF0562	21524	20490	23548	17846	17916	21796	18012	23608	21338	16456	21154	19034
VF0094	30462	28426	34512	19768	22918	28552	19284	28794	27268	17916	23718	20506
VF0467	28628	23236	29554	22438	23008	27154	24498	34082	30094	20478	28714	26522
VF0564	32968	26848	32076	26272	24824	28218	26094	36202	30946	24188	30106	31294
VF0095	31936	25554	33308	26064	27016	31326	26430	34916	31574	23546	32082	28112
CVF315	38080	30278	36036	30458	29532	34684	27830	36096	32198	24742	33384	29656
VF0268	35326	30590	36572	32262	33568	37426	32958	44210	40586	30106	37912	36304
CVF506	54978	50644	56806	50540	48376	53704	49120	63376	55712	44824	58540	57388
VF0272	54386	48312	57372	50986	52064	56806	52046	66798	58680	44050	59810	57760
IA001	112876	95364	120718	83406	92332	107132	78858	106518	98568	74960	95586	87744
VF0108	0	6	0	0	0	0	0	0	0	0	0	0
AI382	2	0	4	4	4	14	0	0	0	2	6	10
VF0244	4864	2476	5252	2234	2070	2560	2406	3756	2820	2678	2726	2280
VF0277	32	82	68	72	78	96	140	74	130	28	108	38
VF0274	10160	9534	9906	8718	8908	10352	8118	10148	9030	6510	8582	7890

VF0091	58958	53848	58978	51364	53130	60854	51398	66354	58674	48018	59318	56754
CVF873	0	0	0	0	2	0	0	4	0	2	2	4
CVF145	12	20	6	4	2	10	0	0	2	4	2	0
VF0231	10	2	28	6	12	6	8	28	18	0	0	0
VF0208	8	4	10	8	14	14	10	14	26	4	18	10
VF0209	4	142	8	0	2	0	0	0	0	0	0	0
VF0120	12	8	42	18	10	12	8	14	32	10	40	8
VF0580	48	6	40	32	12	8	14	10	4	26	88	18
CVF379	32	102	136	46	36	14	24	10	20	18	50	26
VF0088	78	104	96	106	94	114	24	20	24	22	38	18
VF0233	54	48	92	16	88	82	30	164	200	38	38	16
CVF645	48	30	52	92	78	120	70	128	92	80	98	116
VF0109	124	216	116	34	50	74	32	154	146	32	100	40
VF0138	96	64	102	74	58	72	102	230	80	66	140	66
CVF206	174	194	190	162	172	210	70	92	138	94	136	88
VF0148	112	170	96	126	120	104	186	218	192	156	236	104
VF0087	68	430	104	38	40	58	98	820	144	100	90	166

CVF618	404	428	396	518	392	540	346	604	466	324	486	428
VF0034	492	474	690	550	574	626	342	494	542	284	444	408
VF0306	404	492	510	534	554	554	602	1072	772	470	722	840
CVF538	1088	1024	1074	770	936	720	816	990	924	636	918	964
VF0167	1216	964	1312	1050	1044	1006	954	1516	916	890	938	916
CVF110	1682	1214	1798	1338	1226	1160	1216	1776	1354	1318	1594	1228
CVF522	1610	1314	1610	1656	1520	1654	1930	2324	1946	1656	2146	2270
VF0090	1814	1324	1854	1622	1616	1734	2068	2414	1916	1560	1808	2206
CVF779	1596	2034	1830	2010	1890	2080	1876	2986	2450	1812	2224	2394
VF0454	3484	3664	2926	1032	1168	1536	1586	2838	2020	1604	1814	1722
VF0073	3054	2938	3002	2350	2618	2708	2560	3772	2528	2264	2716	2476
CVF322	4248	2612	3320	2226	2292	2582	2784	3304	3140	2372	3372	3090
VF0169	4072	3600	3880	3540	3698	4104	3936	5082	4280	3216	4016	4424
CVF522	5808	5218	5480	4174	3994	4898	3302	5390	4592	3264	4302	3294
VF0455	4892	4258	5214	4464	4848	5714	4418	5786	5058	3474	4480	4286
CVF857	5670	4894	5782	5342	5362	6164	5290	6178	5494	4490	5586	5566
VF0144	7256	5602	6036	5780	5972	6866	5938	7072	6602	5074	6588	5732

VF0074	7076	6272	7240	5942	6396	7086	6630	7758	7006	5494	7426	7124
VF0079	7344	5964	8112	6830	6002	6010	6414	8414	6834	5590	7242	7060
VF0456	7634	6696	8076	6332	6494	7244	7246	9438	7470	5958	7870	8298
VF0457	9598	8288	9220	7576	7460	7954	8002	10410	9686	7570	9536	9744
CVF523	10930	8192	10052	8986	8808	9846	8842	10520	9798	8454	10206	8854
CVF645	12236	10394	12012	11110	11202	13594	10310	12686	10922	8838	11334	10512
VF0171	14056	11126	14516	11910	11876	12834	11032	14318	11796	9742	12872	11624
VF0561	15064	12656	14600	12500	13012	14820	11086	13740	12862	9360	12206	10660
VF0168	15446	12646	14834	13222	12804	13374	13200	17444	14122	11424	14594	15110
CVF854	16878	12928	16160	12670	13098	14854	13606	16976	14612	12658	14956	15694
CVF282	19246	13990	16084	12686	12962	13354	13150	17896	15298	11982	14942	14808
VF0003	17650	13212	18098	14014	14382	15796	15028	17822	15748	12712	15894	15892
CVF186	20376	15860	17772	15750	16166	17920	15134	19452	17862	13082	18542	16648
VF0361	20148	16558	20906	18706	18248	20400	19042	23506	19910	15878	20832	20598
VF0560	26068	24006	29176	20150	20282	22954	21496	29458	24162	19448	23394	23294
VF0072	28318	25026	27874	24674	26140	28228	26896	35026	29626	22010	31098	29774
VF0436	32896	26862	32016	28134	28592	30566	26876	33232	31182	25790	33254	29850

CVF523	35100	31290	36516	32142	32782	36060	31216	40014	36528	27952	36564	35086
CVF760	0	0	2	0	0	0	0	0	0	0	0	0
TX449	0	0	0	0	0	0	0	0	0	0	2	0
SS132	0	0	0	0	0	0	2	0	0	0	0	0
AI132	0	0	0	0	0	0	0	0	0	2	0	0
SS022	0	2	0	0	0	0	0	0	0	0	0	0
SS165	0	0	0	0	0	0	0	0	0	0	0	2
TX450	0	0	0	0	0	0	0	0	0	0	4	0
SS143	2	0	0	2	0	0	0	0	0	0	0	0
CVF019	4	0	0	0	0	0	0	0	0	0	0	0
TX383	0	0	2	0	0	0	0	0	0	2	0	0
SS169	0	4	0	0	0	0	0	0	2	0	0	0
IA006	8	0	0	0	0	0	0	0	0	0	0	0
AI085	0	0	0	0	0	0	0	0	10	0	0	0
AI256	4	0	0	0	4	2	2	0	0	0	0	0
CVF179	0	12	0	0	0	0	0	0	0	0	0	0
CVF483	6	0	0	0	0	0	0	4	2	0	0	0

TX445	0	0	0	2	0	0	4	2	2	0	4	0
SS185	0	2	0	2	8	0	0	2	0	2	0	0
CVF537	2	0	6	0	0	2	2	0	0	0	4	0
SS046	12	0	0	0	0	0	0	0	2	2	0	0
AI107	0	0	2	0	0	4	0	0	10	0	0	0
SS135	0	0	2	0	0	0	0	0	2	0	6	8
CVF301	0	18	2	0	0	0	0	0	0	0	0	0
CVF022	2	2	6	0	0	0	4	0	6	0	0	0
AI131	0	18	4	0	0	0	0	0	0	0	0	0
AI022	4	2	4	0	2	0	4	4	2	0	0	0
AI067	2	2	2	0	2	4	0	0	4	0	4	2
VF0133	0	14	4	0	0	4	2	0	0	0	0	0
SS171	4	2	0	0	0	0	2	10	4	0	2	0
TX418	2	0	0	4	0	0	2	4	2	0	4	6
CVF238	20	0	0	0	0	0	0	8	0	0	0	2
AI049	0	0	10	6	2	0	2	0	0	2	4	6
CVF524	6	0	4	0	0	2	6	4	0	2	8	0

SS130	0	20	2	0	0	10	0	0	0	0	0	0
SS127	2	6	2	14	6	0	0	2	0	2	0	2
AI078	2	6	0	2	2	2	0	8	0	8	4	2
AI071	2	4	6	0	0	2	2	10	10	0	0	0
AI084	0	2	10	0	2	2	0	0	2	0	8	12
VF0043	8	6	8	0	4	2	0	0	2	10	0	0
IA047	6	6	2	0	6	0	4	0	4	2	10	0
SS166	2	2	4	4	0	0	0	0	4	6	16	4
VF0557	2	2	0	0	2	6	4	10	6	0	12	0
AI157	0	0	0	0	10	2	4	0	0	0	20	8
AI119	0	10	4	2	2	0	8	4	6	2	4	2
SS141	0	0	6	6	0	2	0	6	0	10	12	6
TX413	12	4	6	4	4	0	0	2	0	0	16	0
CVF027	4	2	2	10	4	0	6	6	6	2	6	2
VF0360	0	8	0	2	18	2	2	10	0	6	4	0
CVF384	2	2	4	6	0	6	0	4	2	4	12	12
AI439	6	2	12	0	0	18	0	4	0	4	8	0

SS168	0	12	10	16	6	2	0	4	0	6	0	0
VF0359	10	6	8	8	2	6	2	2	2	2	2	8
VF0547	6	0	2	22	2	0	4	10	8	0	4	2
AI126	0	12	8	4	16	8	4	2	2	0	2	2
AI416	0	2	4	2	2	0	24	6	12	0	6	2
CVF024	4	0	8	8	6	10	2	8	10	6	0	4
VF0057	6	2	22	0	2	0	10	4	8	8	4	2
CVF542	0	76	0	0	0	0	0	0	0	0	0	0
AI420	6	58	0	0	2	0	0	0	0	0	4	6
CVF090	4	2	16	14	2	0	6	10	8	8	4	8
AI414	2	4	6	6	6	2	12	14	2	10	0	20
AI415	0	2	2	6	30	8	6	10	0	2	4	22
CVF592	6	0	2	18	8	8	6	8	6	14	4	20
AI244	4	0	8	8	8	8	20	24	4	12	6	0
VF0309	6	4	24	2	8	4	10	6	18	10	6	6
TX170	8	10	2	42	18	22	0	0	4	0	2	0
AI106	12	22	24	2	8	24	8	0	4	0	0	4

AI130	8	0	26	10	6	10	8	2	6	4	24	8
IA057	0	100	6	0	2	0	0	0	2	2	0	0
AI437	2	6	6	2	2	4	6	26	0	12	30	18
SS068	6	10	16	0	8	8	10	12	20	2	20	6
SS131	2	4	14	6	30	28	4	2	8	6	12	2
VF0482	2	16	20	12	38	12	2	4	0	2	10	2
SS045	16	0	90	0	0	0	2	0	0	24	0	0
SS006	8	14	14	30	22	0	16	10	0	16	0	4
CVF604	8	4	14	4	2	4	0	60	38	2	0	0
CVF706	6	2	4	18	0	10	16	22	12	8	30	12
VF0438	10	10	6	28	6	18	6	14	10	6	24	2
AI211	18	4	4	12	12	12	16	22	10	8	14	8
CVF222	4	12	8	6	4	20	8	22	6	14	30	12
CVF178	4	16	20	26	14	4	10	8	18	2	18	8
AI279	66	10	18	4	8	12	6	16	2	8	2	0
SS136	10	8	120	2	0	0	4	2	0	8	0	0
TX416	6	0	8	10	44	16	6	12	0	12	10	32

SS011	8	8	18	20	36	10	2	2	0	26	18	12
VF0170	24	8	74	4	14	16	4	0	8	8	2	0
CVF431	4	94	26	8	4	4	2	10	4	2	10	0
AI014	2	4	18	0	16	44	6	8	16	12	34	12
SS104	14	2	94	0	0	0	10	40	4	4	0	4
CVF030	16	6	30	6	6	12	0	66	36	0	0	0
CVF242	4	4	0	24	16	36	4	34	8	12	36	4
AI047	10	14	18	22	8	24	10	8	14	8	36	10
TX407	16	6	22	22	10	10	16	16	4	12	38	12
SS121	10	16	6	22	12	20	12	36	38	14	4	6
VF0439	14	20	6	10	26	46	18	10	20	12	16	0
VF0232	20	2	30	2	16	26	10	20	34	8	28	2
VF0350	12	8	6	28	6	6	12	34	22	22	18	28
AI199	14	6	38	18	8	16	16	28	24	10	4	22
SS011	6	24	38	10	14	16	20	18	4	12	18	28
AI446	2	26	14	12	66	38	6	16	6	8	12	4
CVF843	10	6	16	12	10	4	50	26	28	22	24	14

CVF243	8	6	24	16	30	18	28	22	10	8	48	12
AI417	10	40	22	46	26	20	4	22	8	8	18	8
CVF473	18	16	6	14	20	30	14	16	44	12	18	26
TX001	12	20	18	14	26	20	12	22	22	18	34	18
SS042	26	62	22	8	20	48	10	34	12	4	2	0
VF0522	20	86	36	36	8	4	8	6	10	10	24	4
TX186	8	16	14	8	8	8	40	76	16	18	12	36
CVF341	30	14	22	38	14	34	10	14	20	12	32	22
SS206	34	28	36	8	42	28	14	8	18	10	14	24
AI058	10	28	38	14	8	32	22	34	36	12	20	12
CVF249	10	2	32	18	18	28	6	22	46	38	34	16
CVF705	64	34	66	26	12	68	6	2	6	4	2	0
VF0157	20	10	22	18	44	44	36	24	32	10	12	30
IA064	70	160	18	0	2	2	4	8	2	8	16	12
CVF589	24	16	24	58	24	34	12	22	54	14	16	16
AI384	34	126	16	0	8	14	2	48	14	2	26	26
VF0433	6	16	8	120	28	30	6	50	36	6	30	4

CVF484	20	10	20	32	22	20	10	88	18	50	24	34
AI229	30	18	66	14	26	48	14	22	32	22	46	22
AI035	22	104	10	32	58	28	16	18	10	10	10	48
TX169	94	84	58	22	18	22	8	12	32	0	8	10
AI353	24	14	16	38	32	28	44	22	52	30	52	30
SS035	22	10	16	30	26	16	42	22	40	28	70	66
CVF844	14	204	18	2	14	60	24	32	10	2	10	2
TX408	20	14	14	46	18	18	46	64	62	44	10	38
VF0556	30	20	16	38	36	48	32	46	64	32	12	30
VF0554	38	34	44	22	34	54	50	20	24	34	38	16
AI339	42	36	46	54	50	44	24	28	14	22	34	20
CVF842	50	16	20	52	22	78	44	10	60	20	38	28
AI445	96	50	44	16	8	12	18	92	20	6	36	44
AI297	22	28	38	58	76	144	26	4	10	8	20	16
AI015	30	26	22	36	38	82	24	32	28	76	24	34
SS058	72	30	136	12	16	60	10	66	32	16	6	2
AI431	54	12	18	26	106	36	18	16	10	14	148	10

SS134	42	44	138	40	44	26	26	24	56	16	10	4
SS005	36	20	56	30	20	12	60	74	68	14	62	30
AI054	28	10	30	44	60	56	46	42	58	30	50	28
VF0052	32	42	36	18	22	34	50	130	52	40	36	30
AI230	18	40	66	42	20	30	26	56	108	30	60	42
CVF539	38	52	58	74	64	62	24	26	24	30	62	28
SS033	32	90	78	50	30	54	24	56	30	10	36	54
AI194	16	340	14	16	8	26	38	2	26	36	16	14
AI104	32	36	22	50	68	38	26	54	66	60	28	74
SS021	46	20	30	38	38	18	50	32	88	46	84	66
AI394	70	48	30	52	24	48	36	76	44	30	44	56
AI361	56	54	44	6	12	54	12	112	118	52	30	14
CVF155	16	18	26	70	42	10	74	102	58	58	70	46
SS114	44	40	30	42	42	100	36	40	58	24	74	66
VF0536	60	124	30	10	18	16	26	196	26	42	34	18
AI143	44	86	40	44	56	24	62	64	50	34	42	58
CVF014	26	36	50	78	64	42	50	86	52	26	68	54

CVF482	46	50	56	50	66	44	68	90	50	40	42	36
AI158	38	44	48	48	50	24	42	48	38	70	76	136
SS061	56	34	18	56	54	166	24	82	62	50	46	32
CVF344	72	48	92	24	32	76	28	90	80	16	90	34
VF0264	26	178	40	38	82	36	26	84	18	42	66	50
AI233	32	122	96	50	34	72	40	74	62	28	36	42
CVF606	38	48	42	74	56	102	40	70	40	56	66	76
AI018	78	46	52	46	26	36	34	80	80	72	88	80
AI424	36	22	70	64	74	26	118	104	44	62	40	64
VF0427	64	80	44	86	72	94	14	120	98	32	12	14
TX033	54	84	48	82	36	74	42	84	74	40	74	38
CVF321	58	22	52	42	56	32	86	112	80	40	58	96
AI396	32	22	18	28	72	24	116	64	66	50	62	186
CVF394	72	30	168	36	22	26	46	122	60	20	106	46
CVF095	108	122	90	36	50	44	32	50	34	66	24	110
SS097	38	448	60	20	32	34	22	44	50	6	10	20
CVF570	42	432	58	26	26	22	30	52	36	10	34	22

SS048	36	78	76	136	52	68	48	72	82	42	68	44
AI159	68	32	104	68	46	86	40	86	116	36	78	46
SS161	60	68	46	102	46	96	52	112	80	42	76	42
AI133	48	52	44	194	76	92	56	70	74	22	72	28
CVF181	76	46	96	26	36	98	74	106	52	58	70	94
TX178	76	70	66	56	54	78	66	72	54	46	78	122
VF0337	68	66	88	72	80	70	48	94	48	60	124	50
CVF028	66	52	96	14	28	94	22	248	234	22	18	8
CVF814	140	58	76	28	56	140	42	62	86	76	88	76
CVF337	200	88	124	42	70	78	52	82	70	48	54	32
IA022	40	44	64	54	68	48	100	102	84	58	88	198
AI137	54	50	116	40	108	40	94	160	84	88	92	32
IA039	72	72	108	54	58	122	74	116	92	46	76	78
AI331	28	298	28	38	88	58	56	90	62	54	58	112
AI449	84	76	180	56	42	108	18	120	130	60	74	58
AI267	66	52	120	52	126	146	76	84	66	94	90	48
AI435	74	82	108	96	66	114	56	80	90	78	138	56

TX177	40	104	60	48	50	20	70	456	78	44	32	36
SS112	70	52	130	90	78	124	64	192	126	28	72	30
CVF761	64	50	94	126	78	160	90	116	90	46	54	90
AI072	110	70	50	70	52	112	52	104	66	130	136	138
VF0413	204	204	146	74	70	50	58	60	62	36	72	72
VF0302	88	52	110	120	110	104	102	54	132	92	106	38
CVF319	188	194	114	54	58	96	66	104	66	58	82	30
AI168	78	50	56	52	44	48	138	164	108	142	118	118
TX171	104	76	114	112	128	162	48	86	120	68	46	64
CVF572	52	36	84	48	66	46	110	142	126	120	86	216
AI108	78	62	84	92	74	72	116	152	82	96	150	104
SS126	78	70	80	46	52	112	58	220	288	86	70	30
VF0285	42	42	176	98	104	100	126	156	128	66	120	62
SS124	106	120	132	98	100	66	96	136	92	94	108	92
CVF291	78	60	154	118	80	138	58	134	106	48	178	94
AI228	56	52	94	126	122	160	116	148	108	72	88	106
AI044	138	88	152	190	120	202	40	96	82	56	40	62

AI099	110	134	94	74	58	64	84	164	162	54	74	196
AI391	158	102	126	136	108	142	104	104	100	42	90	82
AI143	96	26	82	94	126	146	90	180	118	96	124	120
AI195	286	482	188	22	34	36	46	74	84	20	26	10
CVF313	186	212	146	52	90	152	76	108	84	58	110	36
VF0257	112	114	108	112	80	168	120	76	106	102	122	118
CVF758	104	88	130	134	156	152	104	118	84	74	122	86
AI217	412	74	368	0	10	2	58	198	44	136	28	24
AI185	170	144	154	104	126	176	102	68	94	78	114	32
CVF590	70	50	88	100	124	104	104	256	122	72	106	170
CVF222	50	60	48	44	46	60	196	234	160	80	180	232
AI278	72	34	80	62	140	122	110	218	192	98	142	122
CVF381	86	46	114	62	56	98	110	216	194	184	116	146
CVF524	130	104	128	144	202	130	86	102	102	116	90	98
AI184	100	76	104	116	156	230	110	112	94	102	174	66
SS111	176	112	106	152	138	156	68	100	170	110	116	98
AI037	114	108	218	102	74	164	50	200	254	76	132	18

TX175	98	142	172	128	120	116	110	144	124	112	114	194
CVF458	136	162	184	218	164	168	52	88	78	54	166	114
SS162	168	294	100	78	56	148	102	252	96	102	132	78
CVF303	98	110	90	156	110	138	140	160	152	118	128	212
AI277	164	88	140	144	124	96	194	302	178	116	114	70
CVF336	146	168	110	70	154	204	80	136	152	140	218	180
AI155	108	76	152	100	136	164	232	204	116	136	112	242
AI105	92	122	184	126	108	116	130	248	158	122	212	162
SS043	172	124	220	120	102	158	144	146	196	144	130	126
IA051	144	110	130	120	176	172	162	182	204	100	196	134
CVF526	94	88	108	118	138	200	200	216	140	166	194	190
AI100	98	94	84	118	158	134	192	218	190	160	158	254
CVF845	190	114	164	144	164	148	102	260	156	156	184	86
SS128	168	160	182	72	76	138	134	480	158	70	104	148
AI136	326	528	348	68	60	104	42	120	132	40	108	22
AI232	78	402	228	94	100	180	132	190	174	92	178	62
CVF215	384	142	462	42	54	22	166	256	114	176	68	32

VF0491	142	134	240	170	154	170	102	130	130	172	194	192
IA050	174	186	178	152	170	104	146	230	196	90	170	158
IA002	130	322	210	146	84	208	98	244	306	74	108	32
CVF221	170	150	142	174	228	156	178	184	152	122	176	160
AI330	186	188	204	134	168	148	184	230	178	104	142	156
CVF323	130	156	248	122	134	240	102	252	130	106	260	234
AI172	332	160	236	110	208	374	100	204	194	126	148	92
CVF005	130	152	150	232	196	190	178	208	218	158	192	284
VF0311	248	96	304	134	86	320	134	196	310	102	282	114
CVF396	146	198	284	196	152	216	164	176	200	172	342	100
CVF662	190	386	326	144	108	288	102	244	172	156	126	130
CVF762	158	216	174	126	164	180	152	402	212	170	226	236
TX176	238	430	300	178	224	140	112	210	184	108	194	106
SS028	130	180	170	168	230	218	230	302	248	166	186	204
VF0412	230	262	230	214	236	246	200	156	240	122	206	116
CVF340	286	180	398	152	212	228	116	186	224	110	268	112
SS013	204	258	184	188	254	224	158	258	178	140	268	158

VF0057	58	158	84	218	122	98	372	444	254	152	198	328
AI146	220	134	252	166	202	178	248	494	166	206	160	224
AI418	10	28	56	114	46	52	142	258	206	114	272	1356
VF0494	328	154	226	202	216	220	254	270	276	138	194	194
SS215	224	158	184	200	208	164	216	320	272	184	302	320
IA059	176	416	228	188	156	212	242	278	244	188	228	214
CVF527	784	282	290	96	132	100	196	212	126	182	254	132
SS010	384	324	238	444	494	94	336	66	146	38	54	200
IA019	248	296	402	198	168	302	128	256	264	108	356	120
SS187	180	224	262	290	256	228	198	256	276	176	264	238
VF0089	210	316	216	218	182	206	260	236	296	192	320	202
IA060	196	256	292	226	204	204	242	312	244	180	252	292
CVF763	220	240	226	340	236	220	230	346	256	172	234	188
AI058	282	190	340	158	136	250	210	400	264	218	296	298
SS188	158	134	154	212	242	150	332	452	268	224	200	528
SS123	180	222	246	136	212	228	244	564	534	174	130	190
SS205	164	272	240	166	156	214	268	360	326	280	298	364

CVF659	320	232	412	202	178	300	236	270	382	284	182	178
AI399	268	234	294	326	306	258	260	234	340	168	228	278
AI442	206	526	190	206	346	258	204	238	268	238	294	268
IA028	432	664	702	78	86	80	152	336	170	174	210	166
IA058	292	442	252	182	160	278	230	376	252	214	318	262
VF0347	284	306	288	302	312	386	360	234	276	230	252	142
AI077	292	206	282	290	242	188	268	346	262	204	388	408
AI444	158	562	236	222	140	202	210	750	276	174	262	238
AI309	220	242	206	246	336	240	350	382	268	264	292	408
VF0548	184	242	360	274	252	310	238	456	282	224	282	418
AI086	258	200	336	160	194	380	138	714	796	132	162	72
CVF813	240	248	312	208	286	370	200	430	344	246	368	294
VF0442	224	296	298	220	326	290	308	406	366	220	328	324
AI241	276	586	182	382	234	370	214	260	274	242	302	308
AI227	242	528	300	294	222	288	286	306	304	272	308	294
VF0301	270	292	348	290	316	480	258	382	456	184	338	156
TX184	232	150	212	226	206	210	398	380	322	276	492	670

VF0163	224	144	238	404	302	308	356	394	306	314	432	364
CVF003	334	254	414	244	322	444	342	278	450	200	388	206
SS020	380	154	462	298	294	444	220	340	358	324	386	238
CVF399	450	324	334	374	396	338	268	328	306	242	302	262
AI073	88	88	86	134	172	72	534	540	392	300	646	888
CVF234	276	214	232	256	424	480	272	464	464	240	292	328
IA065	274	272	316	218	264	382	366	500	436	256	374	402
CVF297	390	282	476	378	366	488	254	242	332	250	352	290
CVF674	318	286	350	424	434	352	284	308	346	308	346	378
AI149	320	248	194	322	428	390	390	360	456	288	426	344
CVF573	384	408	340	336	380	372	410	412	356	254	376	180
CVF607	324	254	408	310	354	386	318	404	446	350	374	328
CVF279	238	746	334	262	246	164	198	868	248	276	360	372
AI181	372	562	384	378	308	394	254	398	260	300	402	306
VF0555	270	208	326	260	240	238	416	408	500	438	430	658
SS207	292	390	414	354	382	416	388	424	390	264	378	402
CVF812	152	214	148	128	168	130	506	590	406	360	666	1038

AI030	192	150	234	150	244	200	360	698	544	480	578	690
SS217	290	266	360	336	330	436	272	538	494	364	508	364
AI413	348	452	496	350	424	424	298	368	442	324	360	364
SS012	630	496	418	372	352	324	322	374	374	278	392	342
CVF093	450	384	456	512	398	524	384	310	358	296	364	290
CVF378	900	440	1018	88	126	172	272	696	456	444	152	142
CVF248	484	324	472	390	348	664	310	434	506	284	466	240
AI031	424	240	454	370	340	448	322	508	542	420	380	496
CVF393	378	448	480	414	410	458	394	630	454	200	444	258
CVF675	424	482	612	410	356	524	364	442	510	298	406	286
TX409	314	310	366	482	498	420	480	566	584	310	424	390
SS214	350	406	444	484	398	356	362	464	540	390	462	492
CVF536	318	622	464	338	374	356	538	548	512	290	368	422
CVF324	636	230	672	298	314	352	368	556	454	468	368	444
SS096	1718	766	504	218	174	296	206	298	244	216	356	180
AI083	526	412	550	542	498	454	418	410	500	266	468	384
IA054	510	532	618	408	468	482	392	502	424	344	484	326

AI171	556	622	356	414	384	380	472	722	508	338	492	378
CVF859	384	312	506	554	444	456	388	688	520	426	572	404
VF0567	490	354	530	442	432	538	418	710	578	316	514	382
IA004	312	740	542	442	502	508	382	568	460	374	474	472
VF0461	1468	580	588	252	174	302	234	610	528	248	522	306
IA026	486	346	580	492	516	602	426	520	638	404	488	366
CVF349	698	554	738	326	318	264	450	722	414	404	418	642
SS017	562	458	520	454	690	690	476	522	486	280	460	354
AI238	460	1062	430	244	380	1336	338	446	426	172	334	330
CVF326	510	482	646	508	564	722	376	406	486	352	664	320
IA035	534	378	592	474	498	664	364	510	496	446	530	620
AI311	504	388	520	494	546	556	476	674	548	412	476	560
SS180	594	422	724	542	494	548	398	502	364	386	618	602
CVF343	510	452	702	376	376	674	364	614	650	470	636	388
SS212	462	338	416	520	434	496	546	628	580	564	562	700
CVF543	1702	496	1064	118	212	144	380	718	322	422	332	342
CVF615	498	286	586	384	420	472	438	910	778	392	466	668

VF0470	1038	340	1286	142	180	410	252	1036	826	422	286	100
SS216	532	582	604	684	552	586	446	628	504	410	558	410
AI114	692	354	618	362	642	554	482	766	668	506	610	594
CVF223	538	454	720	500	618	618	558	548	672	472	546	654
VF0465	538	564	644	572	508	568	532	940	680	488	640	494
SS117	568	498	818	464	538	544	414	912	938	398	658	440
SS176	830	424	1128	282	282	292	602	866	546	692	654	732
AI180	346	342	416	504	356	490	812	844	712	594	912	1278
VF0051	648	658	558	552	606	758	540	574	726	580	846	664
SS049	684	394	720	594	524	474	696	886	650	550	734	850
SS027	762	472	802	510	638	856	508	730	872	540	792	486
AI387	600	1280	740	656	646	764	472	628	730	554	408	512
IA045	710	956	886	638	640	932	522	588	664	416	918	486
CVF339	610	712	826	664	730	1012	544	710	754	488	840	562
SS189	630	572	744	722	776	954	556	810	826	510	732	724
VF0351	810	598	1014	534	558	678	698	946	704	646	858	786
SS055	1434	520	644	646	646	460	638	986	632	586	856	864

AI153	710	464	754	744	666	792	670	890	750	704	944	892
CVF342	816	648	1038	720	644	1118	502	866	850	432	940	574
AI139	728	494	930	744	846	892	624	1024	890	546	778	684
IA048	802	698	786	604	638	740	690	1222	1138	542	734	814
CVF661	774	794	894	726	634	870	716	886	994	604	808	820
SS210	730	570	862	814	864	918	636	1030	892	712	998	780
IA024	1618	872	1006	826	696	652	654	770	754	582	724	678
SS195	1042	842	1124	692	624	768	796	1100	808	762	712	728
SS024	1844	808	762	508	486	540	686	1002	840	642	890	996
IA056	352	474	380	564	610	468	1206	1338	998	778	1200	1672
CVF840	824	786	1010	1000	954	928	794	880	990	614	866	868
AI310	870	1372	1006	694	632	846	738	980	938	750	956	740
CVF148	798	770	792	948	982	1048	902	996	938	590	1048	830
SS213	904	950	1020	972	890	880	558	1268	916	712	1100	698
AI343	902	2436	574	478	662	608	766	1914	688	436	472	984
AI324	714	710	696	642	778	732	808	1428	1176	986	1140	1210
AI231	670	650	748	810	896	820	984	1296	1172	794	1078	1274

IA020	2370	938	1082	748	606	986	806	878	944	620	1194	736
SS204	826	592	1124	616	740	1000	650	1870	1820	824	962	920
VF0307	978	712	1130	884	996	1094	946	1198	1166	812	1218	1138
SS192	914	888	1008	1024	1048	950	1050	1314	1190	822	1318	870
AI150	928	924	1020	1124	1116	1186	898	1282	1118	920	1018	1114
AI354	1624	1208	1226	832	694	864	968	1348	868	804	1158	1148
CVF670	1224	1148	1240	964	892	1114	866	1342	1150	818	1076	990
AI138	1852	956	2132	608	824	852	786	1582	880	1018	816	594
AI109	868	592	772	928	828	1006	1172	1328	1210	946	1762	1650
VF0308	2562	1006	1858	752	764	708	890	1154	830	924	854	870
CVF223	638	546	718	766	710	982	1590	2836	1474	858	1230	1208
AI118	1286	1104	1142	1126	1226	1136	1044	1302	1204	842	1216	972
CVF287	1218	1002	1058	1004	1132	1042	1134	1300	1288	1018	1202	1370
CVF382	1726	928	1378	762	858	1144	1040	1464	1352	794	1294	1148
AI147	862	728	850	894	974	1060	1392	1450	1206	992	1582	2112
CVF771	1348	2342	1138	1148	1132	1416	930	1252	1208	824	1058	752
SS186	658	572	612	804	1070	878	2162	2250	1526	978	1134	2132

CVF647	1202	1042	1608	1020	1322	1584	1188	1150	1410	1060	1356	926
VF0433	1866	1000	1522	960	1070	1032	1242	1588	1362	1002	1178	1512
IA010	1514	1818	2122	882	886	1398	868	1796	1862	974	1114	704
VF0245	1154	1764	1264	1102	1082	1402	1332	1704	1492	1076	1510	1994
AI315	1256	1024	1432	1234	1260	1418	1356	1852	1830	1356	1542	1630
AI140	1314	1282	1606	1368	1374	1534	1392	1894	1748	1026	1608	1536
VF0151	1338	1236	1498	1396	1428	1606	1636	1702	1624	1150	1606	1846
CVF759	2096	1352	1132	1074	1096	994	1572	2110	1438	1248	1800	2284
CVF338	1392	1734	1996	1146	1148	1928	1104	1990	1746	912	2074	1580
IA036	2008	1936	2494	1298	1360	1674	1110	1816	1398	1194	1340	1298
CVF397	1724	976	1966	1202	1302	1286	1682	2214	2048	1356	1864	1484
CVF286	1550	1716	1684	1742	1456	1872	1562	1894	1858	1128	1488	1278
AI298	1296	1824	1402	1902	2050	2054	2208	1436	1814	950	1266	1204
AI257	3316	1798	2620	1288	1202	1466	1240	1664	1258	1232	1428	1146
VF0366	1650	1592	1716	1524	1542	1610	1268	1866	1668	1702	1846	2200
CVF648	1732	2172	2084	1518	1832	2236	1384	2094	1684	1084	1596	1292
CVF400	1620	1990	1544	1476	1682	1908	1788	2358	1854	1342	1668	1616

AI081	1212	1670	1602	1424	1602	1780	1796	2764	2006	1366	1964	1896
IA043	2826	1516	3244	1146	1092	1224	1386	2442	1990	1802	1250	1232
CVF839	1546	1806	1736	1486	1436	1632	1898	2296	1806	1304	1884	2392
AI215	2482	1596	1700	1572	1644	1896	1766	1976	1762	1246	2020	1874
CVF294	1720	1206	1808	1388	1628	1966	1724	2232	2350	1780	2154	1748
VF0153	2252	2486	1836	1860	1664	1656	1488	2228	1514	1192	2004	1636
IA049	1922	2072	2214	1544	1760	1798	1490	2186	1914	1710	1772	1786
SS164	1924	2656	1828	1976	2174	2758	1476	1468	1594	1466	1646	1258
CVF320	2042	1410	1720	1676	2038	2104	1784	2552	2350	1514	2086	1730
CVF397	1732	1322	2124	1820	1928	2514	1886	2304	2246	1558	1962	1866
IA046	1928	1990	2816	1346	1420	2714	1118	3196	3484	1206	1932	1028
IA062	2486	1870	2588	1488	1668	1778	1686	2770	2156	1730	2018	1940
SS016	1974	2844	2448	1774	2034	2174	1542	2672	1918	1446	1784	1642
SS021	2656	2034	2580	1956	2076	2430	1646	1956	2024	1620	2180	1420
CVF306	2138	1616	2116	1838	1898	2070	1550	2542	2438	1920	2408	2062
VF0469	1730	1956	2146	1592	1514	1766	2060	2684	2740	2068	2308	2954
CVF833	2490	1660	2534	1878	1908	2482	2086	2962	2458	1750	2150	1900

IA025	2890	2900	3324	1742	1580	2120	1772	2654	1918	1962	2150	1682
IA012	1966	2860	2592	1902	1816	2256	1986	2936	2412	1700	2340	2072
VF0396	1924	1820	1882	2228	2138	2280	2392	2654	2366	2000	2502	2744
IA033	2856	4096	2986	1704	1894	2220	1576	2492	2046	1596	1670	1920
CVF383	1930	1484	1886	2178	2270	2580	2410	2988	2782	1826	2480	2544
SS178	2092	1872	2118	2010	2138	2070	2226	3086	2576	1916	2390	3038
AI094	2272	3002	2994	2172	2258	2912	2134	2290	2422	1508	2198	1660
AI116	2298	2238	2392	1896	2050	1976	2356	3278	2512	2164	2498	2744
AI148	2168	1894	2670	2322	2538	2790	2112	2784	2590	2040	2442	2114
CVF660	2368	2248	2712	2066	2246	2490	2702	3372	2748	2158	2910	2756
IA023	2132	2940	2298	2308	2284	2684	2236	3118	2926	1992	3000	2894
VF0472	3164	2438	3670	1920	2210	2758	2318	3360	2964	2024	2138	1904
IA038	3728	2866	3602	2076	2032	2250	1790	3372	2906	2420	2462	2848
VF0141	2528	2302	2752	2142	2296	2336	2610	3434	3444	2626	3120	3258
CVF528	2404	2196	2856	2114	2238	2740	2608	3552	3132	2772	3174	3064
VF0253	3014	3208	3596	2410	2944	2796	2424	3016	2930	2038	2738	2194
VF0124	3228	2942	3242	2436	2284	2732	2476	3420	2886	2420	2652	2754

CVF774	2716	2882	3166	2890	2840	2902	2678	3236	2968	2008	3202	3052
CVF204	2688	2664	2872	2890	3144	3264	2958	3378	2716	2314	2890	2786
CVF363	2698	2668	3114	2574	3020	2856	2884	3444	3114	2242	3234	2776
VF0422	3534	2506	2394	2836	2644	2474	2844	3804	2942	2628	3020	3522
IA041	4086	2964	3992	2332	2086	3108	2176	3814	3716	2050	2654	2182
CVF281	3112	3282	3356	2670	2650	3012	2748	3718	3210	2376	3006	2806
CVF245	3132	2344	3454	3018	2908	3648	2746	3448	3336	2588	3482	2898
CVF834	3186	3180	3136	3204	3320	3528	2940	3470	3188	2342	3198	2792
CVF653	4206	3152	3616	3038	2590	3802	2466	3408	3436	2124	3440	2508
VF0100	3268	2440	3450	3210	3074	3362	2840	3842	3404	2500	3286	3350
CVF838	3464	3280	4210	2720	2394	2964	2530	4120	3178	2918	3376	2970
CVF310	2642	2612	2988	2912	2824	3278	3330	3798	3472	2734	3640	4230
SS023	3088	3130	3426	2676	3032	3298	3034	4216	3424	2714	3436	3452
VF0464	2672	2516	3032	2824	2840	3234	3452	4410	3842	3034	3468	3796
CVF773	3776	3044	4536	2652	2772	2616	2884	4182	3612	3020	2930	3616
CVF587	4320	3370	3994	2970	2848	3054	2996	3912	3184	2650	3694	2790
IA027	4260	3422	3730	2758	2886	3970	2824	3578	3178	2796	3730	3420

IA044	2782	3628	3176	3210	2866	3046	2996	4360	3796	3074	3908	3928
VF0441	4114	2880	4180	3602	3714	3654	3172	3654	3232	2854	3404	3448
VF0449	3278	3772	3880	3286	3238	3604	3396	4620	3924	2890	3976	3858
CVF655	5802	4284	5316	2902	3008	3110	2964	4920	3530	2996	3238	3330
AI368	3434	4772	3580	4090	3792	4028	3396	4506	3654	2746	3620	3788
CVF289	4830	4010	4670	3608	3300	4048	3122	3936	3622	3106	4242	3386
CVF285	4878	3544	4316	3334	3068	3696	3414	4468	4068	3290	4034	4174
SS179	3812	4738	3882	4298	4246	4430	3688	4648	4464	3218	3934	3666
CVF240	3374	3148	4138	3762	3514	3614	4192	5376	4502	3802	5132	5396
SS007	4248	3320	4852	3184	3846	4196	3818	4946	5024	4070	4558	4204
VF0414	4434	3800	4684	4018	4190	4574	4492	5330	4892	3784	4830	4856
IA042	4184	4864	5312	3398	3990	4510	3562	5954	5438	3518	4558	4642
CVF309	4646	4092	4584	4068	4022	4536	4112	5802	4726	3664	5252	4784
AI186	5202	4092	4872	3878	4168	4652	4370	5684	4850	3820	4902	4686
CVF657	5382	3588	3974	3948	3648	4050	4962	5978	5042	3626	5526	5880
IA063	5552	4440	5314	3504	3416	3426	4694	6512	5666	4606	4824	6188
CVF149	5816	4856	5420	4012	4342	4618	4288	5886	4990	4206	5432	4932

CVF591	4964	4604	5296	4958	5510	5480	4624	4974	5134	4128	5628	4620
CVF566	6900	4390	5564	4392	4486	5292	4742	5564	5428	4448	5166	4872
VF0450	13840	6322	8684	3170	3170	3954	3636	5518	4828	4192	4586	3156
CVF396	5452	5542	5726	5546	5572	6168	4954	6508	5674	4328	5374	4598
VF0050	5280	5372	5650	5550	5270	5868	5430	6912	5892	4238	6246	6124
VF0101	5160	4646	5528	5444	5324	5740	5678	7452	6904	5360	6672	6762
CVF834	5740	4822	5330	5192	5024	5736	6220	7488	6342	5138	6842	7758
VF0043	5536	4824	5678	5716	5818	6036	5944	7216	6328	5130	6864	7266
CVF307	5932	5308	6470	5302	5428	5972	6174	7522	6420	5402	6744	6946
CVF300	6362	5754	6662	6212	6330	7090	5500	6882	6680	4786	6066	6108
VF0474	8486	6508	6486	5182	5322	5494	5446	7100	6468	4942	6838	6392
SS025	7178	6436	6362	5472	5726	6168	5420	8140	6470	5066	6666	6574
IA011	7194	6316	8252	6020	6074	7424	5650	7074	6518	4722	6594	5112
CVF043	7916	6200	9150	5774	5452	6838	5394	7968	6404	5702	6032	5292
CVF393	7510	5940	6884	6422	6542	6808	6652	8340	6994	5498	7538	7368
CVF380	7532	5806	7524	6160	6930	8238	5712	7756	7458	5850	7926	6296
CVF494	7196	6300	7234	6450	6350	7064	6486	8526	7504	5938	7682	6868

CVF308	8240	6390	7942	6270	6194	7000	7016	8974	7236	6212	8122	7916
AI169	8294	8062	8156	7028	6370	7014	6928	9124	7664	6170	7532	7764
VF0044	7128	7364	7534	6936	6618	8050	6576	9486	8530	6908	8440	7884
CVF318	7912	5950	7838	7122	7820	8616	7484	10464	9616	6680	8686	7728
CVF153	7784	7246	8410	7678	7526	7772	7618	9774	8924	7070	8754	8098
VF0362	10872	7208	8470	6926	7498	7996	8270	11492	8546	7052	8290	9440
AI098	11490	6870	12824	5852	6518	6572	7372	11820	8776	7722	8434	7956
VF0552	9518	9600	9752	7726	7744	8232	6900	10400	8272	6886	8986	8530
CVF302	8750	8432	8376	8094	8412	9314	7764	10266	9170	7218	8760	8694
AI240	10074	8328	9086	7628	8032	8488	8170	11096	9398	7178	9376	9064
VF0085	7906	9132	8318	8872	8324	8628	8222	11076	9166	7208	9802	10262
CVF305	9918	8654	9944	8562	7776	9410	8458	11458	9628	7788	10516	9682
IA034	8896	8572	10110	8920	8402	9880	8826	11296	10752	7706	9718	9952
IA003	9644	8378	10456	9180	9156	9602	9158	10704	10498	8044	10652	9856
CVF620	10674	8362	10370	8504	8524	9356	9318	12072	10648	8404	10440	11050
CVF311	12516	10450	12114	9744	9912	11162	9696	12694	11054	8936	11440	9856
CVF656	12304	9766	12046	9620	11464	12216	12696	13354	11576	8612	12168	11892

VF0157	12244	10026	12178	10770	10484	11910	11730	14970	14048	11664	14224	14108
CVF536	13246	11140	13348	11024	11466	12932	12208	15836	14038	10632	13206	13536
AI117	13852	12264	13662	11940	12136	12482	11966	15586	13708	10968	13808	13622
CVF652	15642	10976	14926	13702	13466	15228	11610	12620	12412	10216	14146	11250
VF0572	13488	10996	15266	12394	12032	13852	12468	15250	14940	10948	13856	13410
CVF567	14240	11360	14766	12186	12846	14762	12598	15404	13854	10656	13882	12536
CVF495	13080	12954	14942	12472	12978	14004	13046	16324	14388	11430	14346	14288
AI145	18438	14694	22356	8064	9896	10074	11596	21240	15160	12540	12088	12218
VF0489	14548	12862	14784	12872	12930	14026	12978	17280	15218	12382	14996	15158
VF0451	17170	15852	19336	11960	12186	12480	12248	19530	15882	12786	13134	13804
VF0559	17356	12864	16088	13554	13552	14934	13994	16822	15650	12552	16310	14846
AI151	17162	17872	17184	14126	14804	17000	14048	19536	16662	12486	16052	14430
CVF775	21438	18480	17722	14094	15042	15158	14072	18560	16296	12060	17192	15504
VF0367	18984	14196	18366	15476	16296	18520	14980	19120	15956	13166	16946	15152
VF0392	16342	15550	18084	15728	16396	18910	15678	18752	16698	12972	17980	16122
CVF383	16724	13394	16440	14804	15746	16572	16936	21730	18372	14236	19554	19460
VF0568	20502	22142	22774	10922	11868	12592	13846	23142	18790	15914	15598	16474

CVF654	21970	15810	20594	18444	17712	21696	15036	16992	17324	13166	19328	14990
CVF295	18938	16946	18496	19852	18004	19772	15284	19648	17432	13476	18414	19560
AI392	19098	15864	18204	16842	16524	17572	18406	22914	19060	14654	20232	20394
VF0553	18080	17314	19440	17446	17638	19142	17382	21466	20384	15572	18578	18930
CVF588	21164	15824	21034	16940	17320	19156	18788	23484	20790	15280	20386	21180
VF0373	18682	14634	21948	20864	21488	20594	21998	28248	21938	18080	22078	24694
AI149	23436	19240	23428	18940	19480	21804	18360	26024	22564	18156	24096	22022
VF0543	22456	18528	22350	19744	20240	22846	20834	26068	22972	17672	23582	22758
IA009	29134	21530	24716	20772	19580	22308	20680	28140	23954	18188	23722	22760
CVF651	21982	20714	23378	22300	23700	25358	22914	29618	25362	18474	25264	25530
VF0033	27728	21038	25722	23792	24004	26036	24400	29058	26188	20340	27500	25702
AI150	32980	24428	28532	20660	20972	22744	23038	31826	25414	20742	25686	24732
IA030	28158	24250	28710	24596	24034	27888	23180	30090	27722	21092	27446	26016
CVF650	28596	23158	29678	26090	25818	29272	25598	31992	28320	21816	29066	26066
CVF795	29848	24270	31864	26428	26766	29722	26958	33564	30994	23306	30184	28324
CVF658	29562	26162	29904	26412	26210	28698	26994	35416	30652	23906	30638	28536
AI142	31936	30076	34160	24428	23432	25400	24858	38948	29858	26854	29416	28972

CVF288	37044	28414	36686	32662	33500	39506	30040	35834	33634	26124	35296	30560
VF0558	35972	31006	34978	30514	29802	32422	30858	40888	35102	28044	36782	36430
VF0519	38598	37076	39124	29748	31446	34974	32746	43798	37574	29210	37726	35676
VF0432	46522	41362	42432	29968	31530	35678	32892	47474	40008	33172	38214	37678
VF0504	56208	49788	48406	35304	35142	39108	35840	52628	44454	34916	42710	43896
VF0542	48164	38426	48460	39772	42254	46562	42992	54044	47040	37844	50798	48420
CVF649	73856	55568	72642	59598	59784	69040	54438	66370	59178	48188	64768	54724
CVF494	78350	73500	79016	69736	68864	75850	74904	94850	82706	65346	84562	84630
